# The C4 protein of tomato yellow leaf curl Sardinia virus primes drought tolerance in tomato through morphological adjustments

**DOI:** 10.1093/hr/uhac164

**Published:** 2022-07-27

**Authors:** Chiara Pagliarani, Amedeo Moine, Walter Chitarra, Luca Nerva, Marco Catoni, Raffaela Tavazza, Slavica Matić, Marta Vallino, Francesca Secchi, Emanuela Noris

**Affiliations:** Institute for Sustainable Plant Protection, National Research Council, Strada delle Cacce 73, 10135 Torino, Italy; Institute for Sustainable Plant Protection, National Research Council, Strada delle Cacce 73, 10135 Torino, Italy; Institute for Sustainable Plant Protection, National Research Council, Strada delle Cacce 73, 10135 Torino, Italy; Council for Agricultural Research and Economics Centre of Viticultural and Enology Research (CREA-VE). Viale XXVIII Aprile 26, 31015 Conegliano (TV), Italy; Institute for Sustainable Plant Protection, National Research Council, Strada delle Cacce 73, 10135 Torino, Italy; Council for Agricultural Research and Economics Centre of Viticultural and Enology Research (CREA-VE). Viale XXVIII Aprile 26, 31015 Conegliano (TV), Italy; Institute for Sustainable Plant Protection, National Research Council, Strada delle Cacce 73, 10135 Torino, Italy; School of Biosciences, University of Birmingham, Birmingham, B15 2TT, UK; Italian National Agency for New Technologies, Energy and Sustainable Economic Development (ENEA), C.R. Casaccia, Rome, Italy; Institute for Sustainable Plant Protection, National Research Council, Strada delle Cacce 73, 10135 Torino, Italy; Institute for Sustainable Plant Protection, National Research Council, Strada delle Cacce 73, 10135 Torino, Italy; Department of Agricultural, Forest and Food Sciences, University of Torino, Largo Paolo Braccini 2, 10095 Grugliasco (TO), Italy; Institute for Sustainable Plant Protection, National Research Council, Strada delle Cacce 73, 10135 Torino, Italy

## Abstract

Viruses can interfere with the ability of plants to overcome abiotic stresses, indicating the existence of common molecular networks that regulate stress responses. A begomovirus causing the tomato yellow leaf curl disease was recently shown to enhance heat tolerance in tomato and drought tolerance in tomato and *Nicotiana benthamiana* and experimental evidence suggested that the virus-encoded protein C4 is the main trigger of drought responses. However, the physiological and molecular events underlying C4-induced drought tolerance need further elucidation. In this study, transgenic tomato plants expressing the tomato yellow leaf curl Sardinia virus (TYLCSV) C4 protein were subjected to severe drought stress, followed by recovery. Morphometric parameters, water potential, gas exchanges, and hormone contents in leaves were measured, in combination with molecular analysis of candidate genes involved in stress response and hormone metabolism. Collected data proved that the expression of TYLCSV C4 positively affected the ability of transgenic plants to tolerate water stress, by delaying the onset of stress-related features, improving the plant water use efficiency and facilitating a rapid post-rehydration recovery. In addition, we demonstrated that specific anatomical and hydraulic traits, rather than biochemical signals, are the keynote of the C4-associated stress resilience. Our results provide novel insights into the biology underpinning drought tolerance in TYLCSV C4-expressing tomato plants, paving the way for further deepening the mechanism through which such proteins tune the plant-virus interaction.

## Introduction

Rapidly evolving climate conditions are seriously impacting agriculture, increasing the levels of CO_2_ and temperature and decreasing water availability, with heavy fallouts on plant growth and crop yield [1]. Drought is one of the most serious abiotic stresses, negatively affecting the growth and reproduction potential of many crops. Water loss triggers a complex array of defense responses, from stress perception to signal transduction, leading to the induction of specific stress-related genes and the accumulation of metabolites that progressively elicit changes at the cellular, physiological, and developmental levels. To activate drought stress tolerance and minimize water losses, plants need to maintain cell homeostasis by increasing water inlet into cells. Plant adaptation to drought mainly includes accumulation of osmolytes and sugars that reduce water loss at the cellular level, stomata closure with consequent inhibition of photosynthesis and growth, build-up of cell-damaging reactive oxygen species and activation of photorespiration metabolism [2]. Water stress also increases the production of the plant hormone abscisic acid (ABA) that, besides causing stomatal closure, plays an active role in the control of leaf transpiration upon drought and recovery conditions [3, 4]. Such mechanisms are finely tuned by a strict transcriptional reprogramming [5] of several stress-responsive genes identified through transcriptomic and proteomic approaches [6], many of which are transcriptionally activated by ABA signaling [4]. Both type and multiplicity of these responses are strictly influenced by the intensity and timing of the stress, as well as by the developmental stage and genotype of the plant and by the environmental factors responsible for the induction of the stress. From a biological and agricultural perspective, it is important to thoroughly understand the physiological and molecular mechanisms that control plant resilience and acclimation to drought, with the final goal to select crops that are better adapted to future climate conditions, and to develop sustainable tools useful for maintaining or increasing crop productivity.

For its fruit characteristics, tomato (*Solanum lycopersicum* L.) has a worldwide recognized and prominent role in food and food processing, but it is one of the most water demanding crops and is particularly sensitive to drought [7]. Tomato is also affected by a number of pathogenic organisms, among which viruses are particularly harmful [8]. The impact of viruses on tomato cultivation and productivity is expected to increase in the future due to the projected climate change scenario, raising the populations of insect transmitting viruses [9–11]. Begomoviruses are among the most important viruses affecting tomato. They belong to the large *Geminiviridae* family, whose members are characterized by geminate particles enclosing one or two circular molecules made of single-stranded DNA, each of about 2600–2800 nucleotides (nt) in size. *Tomato yellow leaf curl Sardinia virus* (TYLCSV), together with other closely related species, causes the tomato yellow leaf curl disease, one of the most damaging diseases of this crop [12]. The monopartite genome of TYLCSV (2773 nt) encodes six genes partially or completely overlapped [13]. Beside the structural V1 gene encoding the coat protein and the C1 gene encoding the replication associated protein (Rep), definite roles for the other proteins of geminiviruses are still to be fully clarified, such as the case of the small C4 proteins, which are produced by six of the fourteen geminivirus genera, and are now emerging as potent viral effectors, with high variability and versatility [14]. Within begomoviruses, the C4 proteins have been found involved in virus movement [15, 16], suppression of RNA silencing [17, 18], symptom induction [19], promotion of hypersensitive response [20] and hyperplasia [21].

In nature, combinations of virus infections and abiotic stresses can occur, leading to the activation of overlapping or synergistic metabolic pathways with novel and unpredictable effects [10, 22]. Notably, recent studies also revealed that viruses can interfere with the ability of plants to overcome biotic and abiotic stresses, including drought [23]. Following the pioneering work describing that virus infection enhances the resilience of plants to drought [24], further examples of beneficial trade-off of virus infected plants grown under drought stress conditions were reported [22]. These observations raised the concept of viruses “re-warding” plants for their infection, operating through conserved metabolic cross-talks that connect the host response to viruses and drought tolerance [25]. These connection pathways may involve the intervention of small RNA regulation, ABA responses, changes in photosynthetic rates, metabolism of osmoprotective compounds, and salicylic acid (SA)-mediated signaling [26, 27]. Occasionally, investigation regarding viruses that prime drought-tolerance resulted in a decrease of transpiration rate and increase of SA accumulation in an ABA-independent manner [22, 26].

Begomoviruses do interfere with host plant metabolism in both model and crop species, altering the expression of genes that govern cell cycle progression, developmental and defense processes, often triggered through different hormonal pathways [28–31]. In the context of drought-induced responses, transcriptome analyses showed that TYLCSV infection boosts the expression of ABA biosynthetic genes, supporting the increase in ABA levels in virus-infected tissues [31]. More recently, *Tomato yellow leaf curl virus* (TYLCV), a relative of TYLCSV, was reported to mitigate the response of tomato plants to heat stress [32] and to increase drought tolerance in *Nicotiana benthamiana* and tomato [33–35]. The study by [33] also advanced that the TYLCV-encoded C4 protein is the viral determinant conferring such features in *Arabidopsis*, in an ABA-independent manner. However, the physiological performances and the molecular events underlying C4-associated drought tolerance are not deeply investigated.

Based on these observations and in search of direct experimental evidence strengthening this concept, we investigated if the C4 protein encoded by TYLCSV could act as a player in the water stress tolerance processes in tomato, the natural host of TYLCSV. By integrating eco-physiological, morphological, biochemical, and molecular data, we observed that C4-expressing plants transpired less than wild-type (WT) plants already in well-watered conditions. This correlated with a delayed onset of stress-related features, accompanied by a much slower inhibition of gas exchange rates, when plants were exposed to the same drought conditions. These effects coupled with an improved water use efficiency, a slight increase in endogenous ABA levels, and a faster dynamic of post-rehydration recovery. Moreover, we demonstrated that such physiological responses were established in C4-transgenic plants through a transcriptional reprogramming of genes associated with water transport, stress defense, proline and ABA metabolism and through specific anatomical and hydraulic traits. Taken together, our results point out that expression of the TYLCSV C4 protein can prime the adaptability of tomato plants to water limiting conditions.

## Results and discussion

### TYLCSV-C4 over-expression induces drought stress tolerance in tomato plants

**Figure 1:**
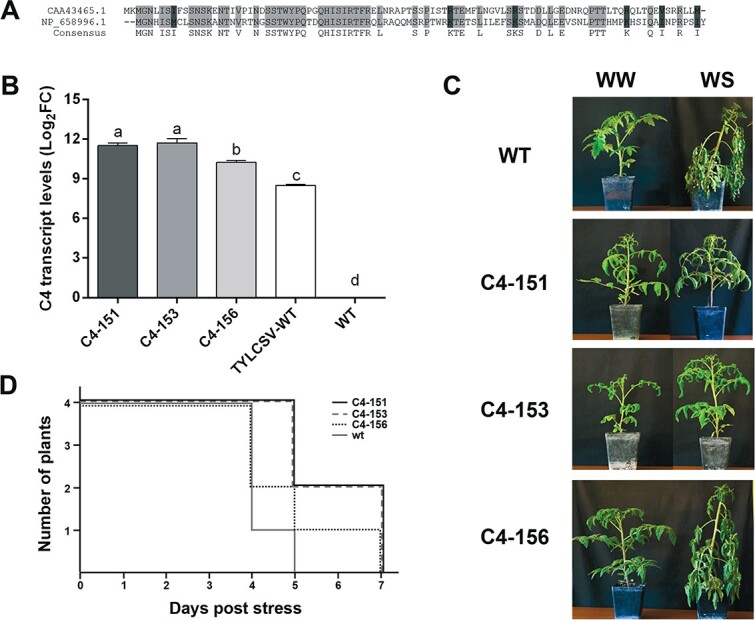
C4 from TYLCSV confers drought tolerance in tomato plants. (A) Alignment of the C4 proteins encoded by TYLCSV (CAA43465.1) and by tomato yellow leaf curl–[Almeria] virus (NP658996.1), with the consensus line showing the identical amino acids; (B) Expression of C4 transcript in leaves of C4 transgenic tomato plants (lines C4–151, C4–153, and C4–156) compared to TYLCSV-infected and healthy WT Moneymaker plants. Transcript levels were normalized using WT healthy plants and the Ubiquitin (*SlUBI*) transcript was used as endogenous reference gene. Three independent biological replicates with three technical replicates for each line and three independent TYLCSV-infected plants (collected at 6 weeks post inoculation) were used for the analysis. Error bars represent SE. Lower case letters above bars represent statistical significance assessed by Tukey’s *HSD* test (*p ≤ 0.05*); (C) Ten-week-old transgenic tomato (cv. Moneymaker) plants (*n = 4*) expressing the TYLCSV C4 gene under the control of the E35S promoter and non-transgenic wild-type (WT) plants were subjected to complete water withdrawal and monitored daily for the onset of wilting. Well-watered (WW) and water stressed (WS) plants were photographed when their collapse was evident; (D) Schematic representation of the time span taken by plants of the different transgenic C4 lines and WT to reach the severe water stress level.

The C4 protein encoded by TYLCSV shares about 48% sequence identity with the corresponding protein of TYLCV (Fig. 1a), which has been recently suggested to contribute to drought tolerance in C4 transgenic *Arabidopsis* plants [33]. To verify if TYLCSV-C4 affects the plant tolerance to drought stress and, in particular, to evaluate if such feature occurs in a crop of industrial relevance, we transformed tomato with the pTOM102NT construct, carrying a TYLCSV DNA sequence (nt 2653–1983) and overexpressing C4 under the E35S promoter. To estimate the basal level of transgene expression in these plants, we measured the C4 mRNA accumulation by qRT-PCR in three independent transgenic lines. Compared to TYLCSV-infected plants, all transgenic lines showed significantly higher amounts of C4 transcripts (up to more than one-third, Fig. 1b). In addition, all transgenic lines displayed similar morphological defects in the leaf shape, which appeared convoluted, crumpled, and downward curled along the major vein, more evidently in the C4–151 and C4–153 lines (Fig. S1).

When we tested the effect of drought stress on these C4 over-expressing lines during a 7-days-long period of complete water deprivation, all C4 plants reached a severe stress condition on average three days later than WT controls (Fig. 1d). However, four days after water deprivation only the C4–151 and C4–153 lines maintained a reduced wilting appearance, while the canopy of C4–156 plants quickly collapsed, similarly to WT plants (Fig. 1c). Accordingly, droughted plants of the C4–151 and C4–153 lines maintained higher stem water potential (*Ψ_stem_*) (*−*1.075 ± 0.03 MPa and *−* 1.16 ± 0.03 MPa, respectively) compared to C4–156 and WT plants (*−*1.24 ± 0.02 MPa and *−* 1.31 ± 0.04 MPa, respectively). Such differential responses to stress among transgenic lines positively correlated with the level of C4 transcript produced (Fig. 1b), suggesting that TYLCSV-C4 can modulate drought tolerance in tomato in a dose-depending fashion.

Therefore, to study the functional role of C4 in regulating the water balance in tomato, we focused all subsequent experiments on the C4–151 line, which displayed stronger resistance to stress and a high level of C4 expression.

### C4 improves the physiological performances of transgenic tomato plants during drought and recovery

To better investigate the effect of C4 on the physiological responses upon drought, we designed a new experiment where gas exchanges and stem water potential were monitored in water-stressed (WS) and well-watered (WW) C4–151 and WT plants. The physiological performances were further inspected considering the dynamic of gas exchange recovery following stress relief (i.e. after rehydration).

**Figure 2:**
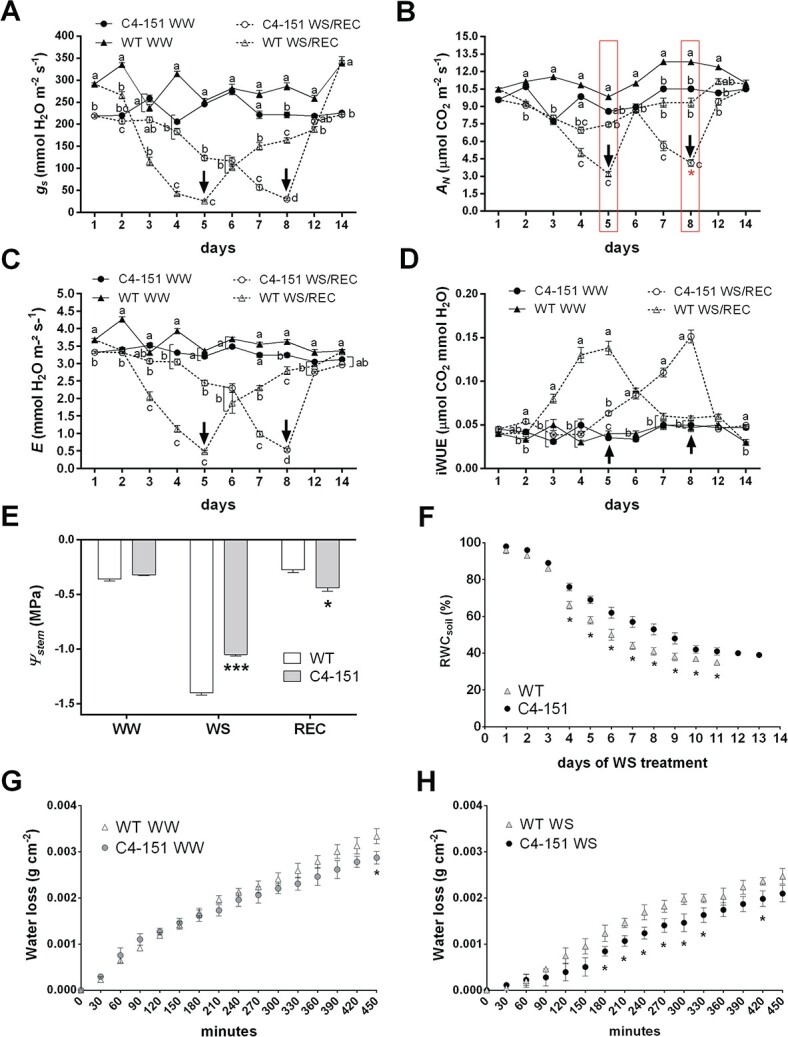
Effects of C4 expression on tomato physiological performances during a drought and recovery time-course. Dynamic changes in the rates of (A) Stomatal conductance (*g_s_*, mmol H_2_O m^−2^ s^−1^), (B) Assimilation (*A_N_*, μmol CO_2_ m^−2^ s^−1^) and (C) transpiration (*E*, mmol H_2_O m^−2^ s^−1^), and (D) water use efficiency (iWUE μmol CO_2_ mmol^−1^ H_2_O) of WT (Moneymaker, triangles) and C4–151 plants (circles) under well-watered conditions (WW, black-filled symbols) and water stress followed by recovery (WS/REC, empty symbols). Water withdrawal started at Day 1, while recovery started following plant irrigation at Days 5 and 8 for WT and C4–151 plants, respectively (black arrows). (E) Stem water potential (*Ψ_stem_*) of WT and C4–151 plants measured under different water conditions (WW = Day 1; WS = Day 5 and Day 8; and REC = Day 14 of the time-course reported in a to d panel). (F) Changes in soil relative water content (RWC_soil_, %) monitored over the whole duration of a WS treatment carried out on a separate group of potted WT and C4–151 plants. (G-H) Water loss in the dehydration assay normalized on a per leaf area unit basis (g cm^−2^) and performed on detached leaves from WT and C4–151 plants grown under (G) irrigated and (H) drought conditions. In panels (A-D), lower case letters indicate significant differences among the four tested conditions within each experimental day, as assessed by Tukey’s *HSD* test (*p ≤ 0.05*), while the red asterisk, when present, denotes significant differences between WT and C4–151 tomatoes at the end of the WS treatment (ie. day 5 vs day 8, as highlighted by the red square) as determined by a two-tailed Student’s *t* test (^*^p ≤ 0.05). In panels (E-H), the asterisks denote significant differences between genotypes under the same condition, as determined by a two-tailed Student’s *t* test (^*^p ≤ 0.05, ^***^p ≤ 0.001). In all panels data are the mean ± SE (*n = 7*).

Over the whole duration of the experiment (14 days), WW C4–151 plants showed on average lower values of stomatal conductance (*g_s_*), assimilation (*A_N_*) and leaf transpiration (*E*) compared to WT (Fig. 2a-c), thought with no significant difference in terms of water use efficiency (iWUE, Fig. 2d). Moreover, under normal irrigation conditions, no discrepancy in stem water potential occurred between the C4 and WT genotypes (*Ψ_stem_*, WW controls, Fig. 2e).

Three days after the beginning of the treatment, WT plants underwent a steep decrease in *g_s_*, *A_N_*, and E reaching at day 5 levels of gas exchanges (Fig. 2a-c) and *Ψ_stem_* (almost −1.5 MPa; Fig. 2e) associated to severe water stress in tomato [36]. Conversely, C4 expression significantly delayed the progressive inhibition of gas exchange rates, as well as the sudden drop in *Ψ_stem_* normally occurring in response to water limitation. Accordingly, *g_s_* rates of C4–151 plants followed a much slower and gradual decrease, reaching levels close to WT plants almost 3 days later (day 8, Fig. 2a). Interestingly, although at day 8 C4–151 plants experienced limiting *g_s_* and *E* values not significantly different from WT controls (i.e. 30 ± 2.79 *vs* 26 ± 2.28 mmol H_2_O m^−2^ s^−1^ for *g_s_*, and 0.52 ± 0.03 *vs* 0.48 ± 0.04 mmol H_2_O m^−2^ s^−1^ for *E,* respectively), their photosynthetic rates (i.e. 4.14 ± 0.23 and 3.18 ± 0.26 μmol CO_2_ m^−2^ s^−1^ for C4–151 and WT plants, respectively, Fig. 2b) and *Ψ_stem_* values (i.e. -1.05 ± 0.014 *vs* − 1.4 ± 0.02 MPa for C4–151 and WT plants, respectively, Fig. 2e) were significantly higher than those of WT plants at day 5. Trends of gas exchange and *Ψ_stem_* measurements were confirmed during the second physiological time course (Fig. S2), highlighting a time lapse of four days between the end of WS treatment in WT plants (day 7, corresponding to *Ψ_stem_* of −1.45 ± 0.05 MPa, Fig. S2e) and the end of WS treatment in C4–151 plants (day 11, corresponding to *Ψ_stem_* of −1.11 ± 0.01 MPa, Fig. S2e).

Compared to WT plants at the end of the WS treatment, C4–151 plants also showed a slight, although not significant, improved water use efficiency (iWUE, Fig. 2d); such difference was more evident and statistically significant in the second experimental trial (i.e. 0.071 ± 0.016 *vs* 0.11 ± 0.018 μmol CO_2_ mmol^−1^ H_2_O, Fig. S2d).

Differences between WT and C4–151 plants also occurred in the recovery dynamics after rehydration. Specifically, at day 14 (i.e. 9 and 6 days after re-watering for WT and C4–151 plants, respectively), the *Ψ_stem_* of both genotypes restored to levels comparable to the pre-stress conditions (recovered samples, REC, Fig. 2e). Particularly, while in the first experiment *Ψ_stem_* of REC C4–151 plants was significantly lower than REC WT plants (Fig. 2e), in the second trial no significant differences in *Ψ_stem_* values were noticed between the two genotypes at the end of recovery (Fig. S2e). In terms of gas exchange recovery, WT plants reached *g_s_*, *A_N_* and E values similar to those of the corresponding WW controls in almost 10 days (day 14, Fig. 2a-c), whereas C4–151 plants completely recovered to pre-stress gas exchange values in only 4 days (day 12, Fig. 2a-c), following a trend consistent with the second experiment (Fig. S2a-c).

To strengthen the observation that C4–151 plants depleted water by transpiration more slowly than WT controls, we gravimetrically monitored changes in the soil relative water content (RWC_soil_) of pots containing either C4 or WT plants, during the whole experiment until canopy collapse (Fig. 2f). The results evidenced that RWC_soil_ values of C4–151 plants were significantly higher than WT plants already 4 days after treatment imposition; moreover, water loss dynamics were delayed in C4–151 plants (i.e. the experiment ended at day 11 and at day 14 for WT and C4–151 plants, respectively) (Fig. 2f), in agreement with the dynamics of gas exchange (Fig. 2a-c). In parallel, a two plants per pot approach was also followed (Fig. S3), allowing us to confirm genotype-dependent changes in RWC_soil_ in a condition where the roots of both genotypes were subjected to the same water stress level. Differences in stress perception were evident both in terms of visual inspection (Fig. S3a, b), as only the WT plant showed a severely wilted phenotype, and in terms of *Ψ_stem_*. In fact, although with the “two plants per pot” approach soil water depletion occurred more slowly than with “one plant per pot” (Fig. S3c), again WT tomato reached *Ψ_stem_* values significantly lower than C4–151 plants (Fig. S3d), mirroring the results obtained in the single plant per pot experiments.

Dynamic changes in leaf transpiration were further deepened by leaf dehydration assays under controlled conditions, monitoring the water loss of detached leaves of WT and C4–151 tomatoes collected from plants under WW and WS conditions (Fig. 2g-h). Overall, leaves of C4 expressing plants tended to lose less water than WT leaves, though differences between the two genotypes were statistically significant only at the end of experiment (450 min, Fig. 2g). Interestingly, differences between the two genotypes were particularly evident already after 120 minutes for leaves from WS plants (Fig. 2h).

Collectively, these results strongly support the hypothesis that the reduced basal transpiration rates of C4 plants is the basis for their ability to tolerate drought stress. The lower stress perception of C4–151 plants compared to WT controls most likely results from a slower response of guard cells to changes in water availability, allowing to more efficiently limit excessive water loss during stress.

Further indications supporting the notion that C4 plants can better counteract the WS effects were searched by evaluating the chlorophyll content under well-watered and water stressed conditions. At the beginning and at the end of the trial (Fig. S4, a and b, respectively), no statistically significant differences were detected between irrigated plants of both genotypes. Although drought significantly increased the chlorophyll content index (CCI) in both genotypes, WS plants expressing C4 displayed a significantly higher CCI than WT (Fig. S4b), possibly associated to their improved performances upon drought treatment. Notably, the results collected during the second experimental trial (Fig. S4, c and d) indicated that, unlike WT plants, C4–151 leaves had significantly higher CCI values already in well-watered conditions (Fig. S4c), a feature that might be helpful for a C3 crop, such as tomato, to better withstand climate changes and extreme drought events. This genotype-dependent effect on CCI was also confirmed in WS plants of both genotypes at the end of the treatment (Fig. S4d). Interestingly, the C4 protein of the geminivirus *Tomato leaf curl Palampur virus* was shown to interact with host proteins involved in photosynthesis [37].

### C4 expression induces differential ABA and proline accumulation associated to changes in the expression of stress-responsive genes during drought and recovery

**Figure 3:**
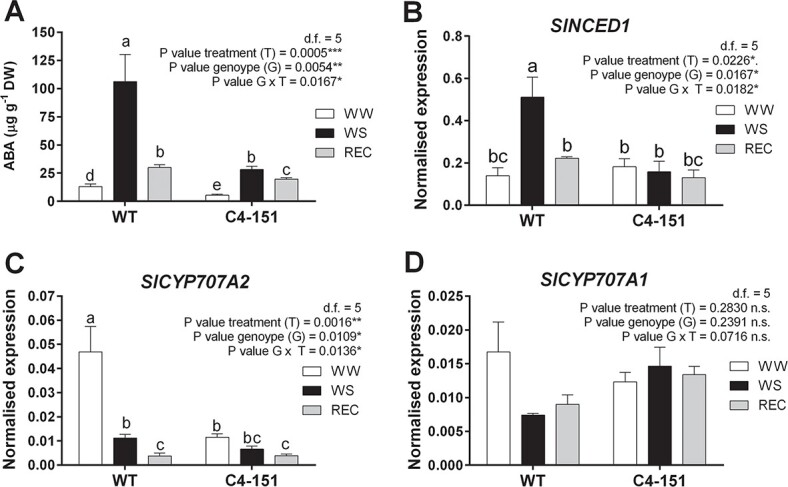
Focus on ABA metabolism. Content of (A) abscisic acid (ABA) and expression profiles of key genes involved in ABA (B) biosynthesis (*SlNCED1*) and degradation (C, D) (*SlCYP707A1*, *SlCYP707A2*) analyzed in leaf samples taken from WT and C4–151 tomato plants subjected to water stress treatment (WS) and recovery (REC), in comparison with well-watered controls (WW). Ubiquitin (*SlUBI*) and Elongation factor 1α (*SlEF*) genes were both used as endogenous housekeeping controls for the normalization of transcript levels. Significance of genotype, treatment, and genotype × treatment (G × T) interaction was assessed by Tukey’s *HSD* test for *p ≤ 0.05* (^*^), *p ≤ 0.01* (^**^), *p ≤ 0.001* (^***^), and *p ≤ 0.0001* (^****^) and the corresponding results are given above each graph in the figure panel; n.s. = not significant. Lower case letters above bars are reported when the genotype × treatment (G × T) interaction and/or genotype (G) main effects are statistically significant as attested by Tukey’s *HSD* or Student’s *t*-test, respectively. Error bars represent SE. Three independent biological replicates with three technical replicates each were used for the analysis.

In search of biochemical and molecular mechanisms underlying the C4-induced drought avoidance or tolerance, we investigated the role of ABA, a plant hormone traditionally considered a primary regulator of the drought stress response [38]. A previous study reported that in TYLCV-C4 expressing *Arabidopsis* plants, ABA content increased following water withholding, not significantly different from WT plants [33]. Similarly, no changes in the expression of ABA related genes were reported, accompanied by contrasting responses of WT and C4 *Arabidopsis* to exogenous ABA application, leading the authors to conclude that C4 promotes plant’s drought tolerance through ABA-independent mechanisms [33]. Conversely, in our survey endogenous ABA levels in C4–151 plants were significantly (almost four times) lower than in WT plants already in WW conditions (Fig. 3a). Such differences were maintained in response to drought, as tomato C4 plants accumulated up to four times less ABA compared to WT (Fig. 3a). The effect of the genotype × treatment interaction was indeed statistically significant (Fig. 3a). As expected, ABA levels significantly decreased during recovery in both genotypes, though they reached a lower value in C4–151 compared to WT individuals (Fig. 3a). Accordingly, the transcriptional profile of the ABA biosynthetic gene *NCED1* followed a trend similar to the ABA level in both genotypes. While *NCED1* transcripts were overexpressed during drought and significantly downregulated upon recovery in WT plants, no changes occurred in C4–151 plants subjected to WW or WS treatment (Fig. 3b).

To gain a more complete overview on ABA metabolism, transcripts encoding two hydroxylases (*SlCYP707A1* and *SlCYP707A2*) involved in ABA degradation [39] were also analyzed. The profile of *SlCYP707A2* in C4–151 plants was consistent with the levels of ABA measured in the same samples, with no significant variations. Conversely, in WT plants *SlCYP707A2* was highly activated exclusively in the WW conditions, with a trend opposite to *NCED1* (Fig. 3c). Similarly, the transcript amounts of *SlCYP707A1* were higher in C4–151 *vs*. WT plants during drought and recovery, though in this case the effects of treatment, genotype, and their interaction were not statistically significant (Fig. 3d).

**Figure 4:**
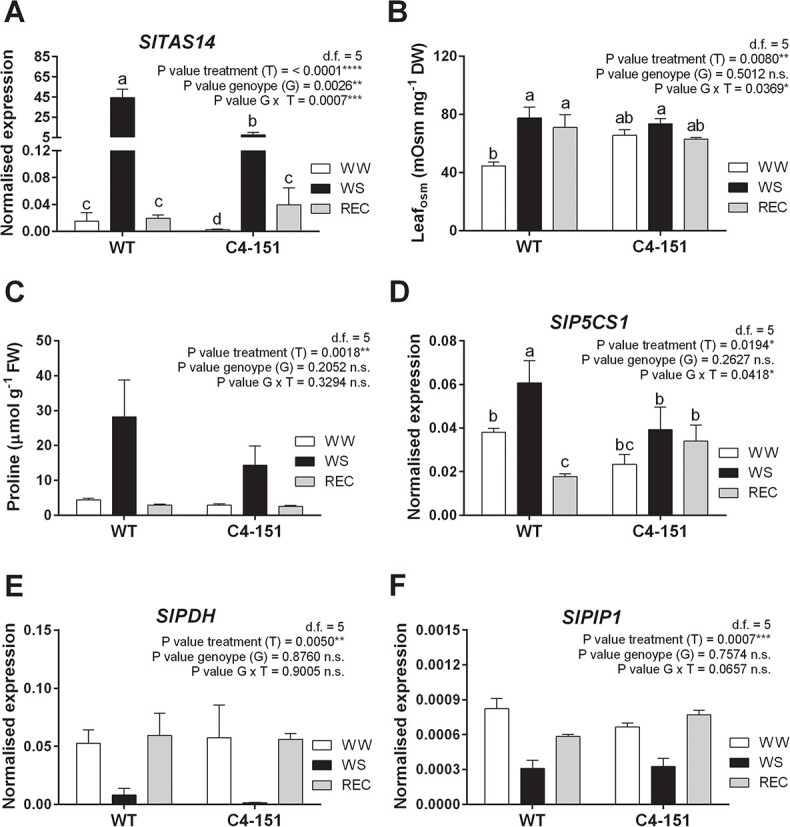
Focus on stress-responsive genes and osmolytes. (A) Transcriptional profile of the key stress-responsive gene dehydrin (*SlTAS14*) analyzed by RT-qPCR, (B-C) changes in (B) leaf osmolality (Leaf_osm_, mOsM mg^−1^ DW) and (C) leaf proline content (μmol g^−1^ FW), (D-F) transcriptional profiles of genes involved in proline (D) biosynthesis (*SlP5CS1*) and (E) degradation (*SlPDH*), and (F) water transport (*SlPIP1*), analyzed in leaf samples taken from WT and C4–151 plants subjected to water stress treatment (WS) and recovery (REC) in comparison with well-watered controls (WW). Ubiquitin (*SlUBI*) and Elongation factor 1α (*SlEF*) genes were both used as endogenous housekeeping controls for the normalization of transcript levels. Significance of genotype, treatment, and genotype × treatment (G × T) interaction was assessed by Tukey’s *HSD* test for *p ≤ 0.05* (^*^), *p ≤ 0.01* (^**^), *p ≤ 0.001* (^***^), and *p ≤ 0.0001* (^****^) and the corresponding results are given above each graph in the figure panel; n.s. = not significant. Lower case letters above bars are reported when the G × T interaction and/or genotype (G) main effects are statistically significant as attested by Tukey’s *HSD* or Student’s *t*-test, respectively. Error bars represent SE. Three independent biological replicates with three technical replicates each were used for the analysis.

Although we cannot exclude a differential regulation of stress responses mediated by ABA in tomato and *Arabidopsis* plants expressing C4, the association of TYLCSV-C4 expression and reduced accumulation of ABA upon drought, together with the lack of statistical differences in the levels of *NCED1* and ABA hydroxylases suggest that the improved physiological performances of C4 plants under water deprivation could rely on factors other than ABA. Additionally, a different response to drought mounted by C4–151 plants was gathered when key genes associated to water stress and ABA signaling were considered. The expression trends of *TAS14*, a dehydrin-encoding gene used as marker of drought stress response [36], typically activated in tomato following ABA treatment [40], were up to 8-fold lower in C4–151 than in WT plants upon drought (Fig. 4a).

To further deepen this subject, we determined on the same samples leaf osmolality (Leaf_osm_) and proline content, one of the major osmolytes accumulated to limit excessive water loss [2] (Fig. 4b, c). In WW conditions, the Leaf_osm_ values were higher in C4–151 than in WT plants, with no significant changes during drought and recovery (Fig. 4b), suggesting that a higher basal supply of osmoprotectants and ions in transgenic plants could support their slower water depletion dynamics (Fig. 2).

Unlike ABA, no differences in proline content between C4–151 and WT plants in WW and REC conditions were noticed (Fig. 4c). Conversely, during water stress, proline levels of the C4–151 line were half of WT tomatoes (Fig. 4c). Noteworthy, proline accumulation significantly increased following WS in both genotypes, though at different extent. Since proline increase upon drought stress can result from either induction of biosynthetic genes or inactivation of catabolic genes [41], we profiled transcriptional changes of the proline biosynthetic gene delta 1-pyrroline-5-carboxylate synthetase (*SlP5CS1*), a homolog of *Arabidopsis P5CS1* [42] induced upon dehydration trough ABA-dependent and -independent signaling pathways [41]. Indeed, upon drought stress, *P5CS1* transcripts of WT plants significantly increased of about one third, whereas only a weak non-significant upregulation occurred in C4 tomatoes (Fig. 4c), mimicking the lower proline levels (Fig. 4b). Notably, while proline concentrations reached pre-stress levels in REC plants of both genotypes, *P5CS1* activation did not significantly differ from WS plants. Conversely, the expression of *SlPDH*, encoding a rate-limiting proline dehydrogenase, activated following proline accumulation during stress recovery [43], and of the drought-responsive aquaporin gene *SlPIP1* [36] were not influenced by the genotype. Indeed, their transcriptional profiles were significantly downregulated upon drought stress imposition in both genotypes (Fig. 4, c and d). We thus reasoned that, while in WT plants the higher proline accumulation under drought stress may result from the concurrent activation of *P5CS1* and downregulation of *SlPDH*, in WS C4 plants, the lower proline concentrations could result from the inhibition of the catabolic gene.

Collectively, these observations suggest that molecular and biochemical signals typically associated with stress defense responses are either differentially regulated or attenuated in C4–151 plants. Such condition may underpin delayed eco-physiological responses of C4 plants, resulting in a slower inhibition of gas exchange rates, also supporting the more rapid and efficient recovery of C4 plants from drought stress conditions.

### C4 expression affects plant morphometric features, but not stomatal density.

To evaluate the involvement of other triggering factor(s) conferring improved drought-adaptability of C4 plants, we investigated morphometric and anatomical features of C4–151 plants. The combined effect of C4 expression and drought treatment on plants was first evaluated by measuring the size and number of leaves, together with plant height and stem diameter, immediately before and at the end of the drought stress treatment, therefore comparing WW and WS plants of both genotypes. While the number of leaves was similar in both genotypes, with no changes following stress occurrence (Fig. S5, a and b), both plant height and stem diameter, measured at the bottom, medium and top of the plants, significantly varied between C4–151 and WT plants (Fig. S5, c and e). These differences were still evident at the end of the experiment, both in WW and WS individuals. Notably, the genotype was the only factor that significantly affected both parameters (Fig. S5, d and f), as water deprivation only influenced the top stem diameter, which increased significantly in WS plants, independently of the genotype (Fig. S5f). All measurements and results were overall confirmed in a second independent experiment (Fig. S6).

Furthermore, as the root system architecture affects water uptake and hence water stress responses, we analyzed the roots of C4–151 and WT plants in both WW and WS conditions, at the end of the water stress time course (Fig. S7). As expected, based on the reduced plant size, both root area and root biomass of C4 expressing tomatoes were significantly reduced than in WT plants. Remarkably, while the WS imposition did not significantly alter the root parameters in C4–151 plants, the root area of WT plants slightly, but significantly decreased.

**Figure 5:**
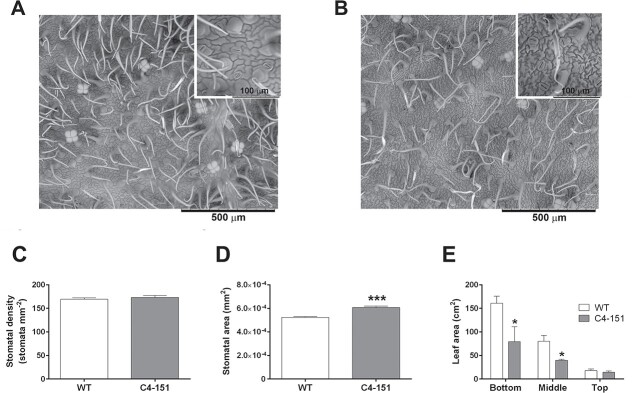
Morphological observations of stomata and leaves. Representative images of stomata from the abaxial leaf surface of (A) WT and (B) C4–151 tomato plants obtained by Scanning Electron Microscope (SEM) observations of intact leaves of the two genotypes. The inset in each image shows a magnified detail of stomata. Data of (C) stomatal density, (D) stomatal area, and (E) leaf area of WT and C4–151 plants represent the mean ± SE (for C, n = 21, for D n = 210, for E n = 7). When present, the asterisks denote significant difference between genotypes, as assessed by a two-tailed Student’s *t* test (^*^p ≤ 0.05; ^***^p ≤ 0.001).

Then, we questioned if alterations in the number and size of stomata and/or differences in the leaf area could play a role in the regulation of C4–151 responses to water stress. Changes in stomatal density and size associated with modifications of other morphometric features, including leaf number and/or leaf/root area can indeed affect the plant’s water relations (e.g. stomatal conductance and transpiration), therefore influencing the regulation of iWUE, especially upon water deficit [44]. SEM observations of the abaxial leaf surface (Fig. 5, a and b) did not reveal statistically significant differences between the two genotypes in terms of stomatal density (Fig. 5c), although C4–151 stomatal area was significantly larger than WT controls (Fig. 5d), a feature possibly compensating the lower stomata number. Furthermore, although differences were statistically significant only for the bottom and medium branches of the plant canopy, C4–151 plants had a smaller leaf area than WT (Fig. 5e). These data, associated with the previously reported morphometric measurements, imply that the improved drought tolerance of C4–151 plants might simply rely on their reduced size. Nevertheless, since C4–151 tomatoes have similar number of leaves and stomatal density and, notably, have much larger stomata than WT plants, one would expect similar gas exchange levels in well water conditions for both genotypes. In this case, transpiration dynamics during drought spell should be similar for C4–151 and WT plants. Such hypothesis was also strengthened when stomata on the adaxial leaf surface were measured (Fig. S8), revealing that C4–151 plants had significantly less but larger stomata than WT plants (Fig. S8).

However, the gas exchange rates during the water stress time course and, particularly, the measurements of soil water loss over time and the leaf dehydration assays (Fig. 2f-h, and Fig. S3) demonstrate that C4–151 plants have an improved ability to limit excessive water loss.

**Figure 6:**
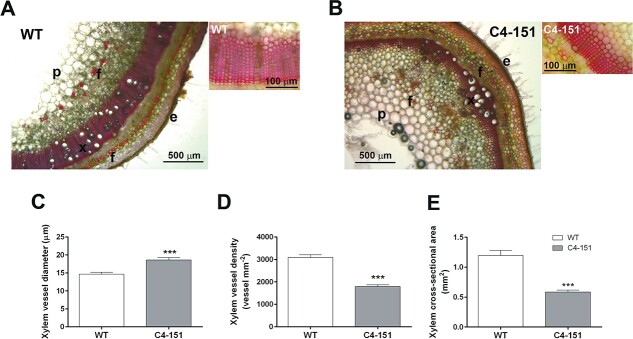
Analysis of stem xylem area of WT and C4–151 plants. (A-B) Representative safranin-stained cross-sections of stems collected from well-watered (A) WT and (B) C4–151 plants; e: epidermis, f: fibers, p: parenchyma, x: xylem; the staining with safranin allows to highlight only the portion of functional xylem conduits. The inset beside each image shows a magnified detail of xylem conduits; (C-E) Values of (C) xylem vessel diameter, (D) xylem vessel density, and (E) whole xylem cross-sectional area calculated by analysing the microscope images [see an example in (A)] with the ImageJ (1.46r, NIH, https://imagej.nih.gov) software. Asterisks denote significant differences attested by a two-tailed Student’s t test (^***^p ≤ 0.001); (*n = 12*).

We therefore reasoned that other intrinsic features should underpin the physiological responses of C4–151 plants to water stress. Accordingly, we observed that C4 expression does not impact plant architecture in terms of leaf formation, but rather it influenced other morphological features, i.e. plant height, stem diameter, root surface area, potentially related to a different size/organization of vascular tissues. Recently, xylem patterning defects induced by TYLCV C4 were reported in seedlings of transgenic *Arabidopsis* plants [45], possibly resulting from the interaction of C4 with the intracellular domain of the receptor-like kinases BARELY ANY MERISTEM 1 (BAM1) and its homolog BAM2 [18]. To assess if alterations in xylem patterning also occurred in tomato plants expressing TYLCSV C4, we analyzed transversal stem sections with a light microscope (Fig. 6a, b). These observations revealed that, although the xylem vessel diameter of C4 plants was bigger than in WT individuals (Fig. 6c), the vessel density was almost half (Fig. 6d), in turn resulting in a significantly narrower whole xylem area (Fig. 6e). These hydraulic adjustments can thus imply an overall lower xylem water transport capacity of C4–151 compared to WT, possibly supporting the different water loss dynamics of the two genotypes (Fig. 2). Additionally, it was demonstrated that structural differences in size and number of stem xylem vessels, combined to specific leaf traits, can influence the ability of plants to counteract water stress [46]. Particularly, a reduction in stem xylem vessel transectional area was reported to facilitate water transport and to limit the plant vulnerability to severe drought effects, also preventing the risk of embolism formation [47]. We therefore reasoned that such anatomical traits could affect the hydraulic conductivity of xylem in C4 plants, likely hindering their basal transpiration rates already under normal watering conditions and consequently improving their responses to water stress.

### C4 expression influences auxin accumulation and signaling

Based on the morphometric results and considering the prominent role of auxin (Indole-3-Acetic Acid, IAA) in vasculature patterning and leaf morphology, we inspected the content of this hormone together with the expression of key genes positively (*SlARF5*, *SlARF8*) or negatively (*SlIAA4*, *SlIAA9*, *SlIAA14*) regulating the auxin signaling pathway [48, 49]. The IAA accumulation pattern was similar in both genotypes, showing a steep decrease in IAA accumulation upon recovery. Nonetheless, IAA concentration was significantly lower (more than half) in C4–151 than in WT plants (Fig. S9a). The transcriptional levels of the auxin responsive factors *ARF5* and *ARF8* followed a trend similar to the IAA content in WT, but not in C4–151 plants (Fig. S9, b and c). Although the profiles of the negative regulators *IAA* genes were not inversely correlated with the IAA content as expected, they were differentially reprogrammed upon the treatments in C4 and WT plants. In particular, in C4–151 plants the *IAA4* gene was transcriptionally upregulated in REC compared to WW and WS conditions, while in WT tomatoes, the same gene was overexpressed in irrigated plants), attesting a significant effect of the genotype × treatment interaction (Fig. S9, d and e).

Auxin homeostasis is vital for plant growth and virus infection and certain viral-encoded proteins were even shown to remodel auxin biosynthesis and downstream responses to promote symptom development [50]. In the case of begomoviruses, transcriptional repression of the auxin signaling pathway following TYLCV infection has been reported for *N. benthamiana* [51]. In addition, the bipartite begomovirus *Tomato leaf curl New Delhi virus* (ToLCNDV) was found to disrupt IAA signaling through the interaction with Tryptophan amino transferase1 (TAR1)-like protein and CYP450 monooxygenase, resulting in reduced auxin content and development of typical viral symptoms [51]. Interestingly, ectopic overexpression of the ToLCNDV-C4 protein in transgenic tomato led to reduced auxin levels and to the down-regulation of *ARF8* expression, mediated by the increase of miR167 [51]. Recently, several tomato ARFs were found differentially regulated in tomato plants during biotic and abiotic stress responses [53]. Indeed, while in WT plants *SlARF*5 and *SlARF*8 appeared down regulated in response to drought [53], no substantial changes in their expression occurred in C4–151 plants following water deprivation (Fig. S9) and plants accumulated on average much lower levels of IAA (more than half) compared to WT plants. Therefore, since TYLCV infection led to the down regulation of different ARFs, including *SlARF4*, *SlARF5*, *SlARF6A*, *SlARF8B* and *SlARF9A* [53], it is plausible that C4 plays a role in orchestrating auxin regulation during infection and in response to abiotic stress.

## Conclusions

Viruses, obligate pathogens requiring living organisms for their survival, profoundly modify the biological processes of their hosts during infection, and co-evolution events, balancing virulence and host survival responses, have been evoked to guarantee infection [23, 54]. Beneficial effects exerted by viruses on their hosts are under investigation, opening the perspective to identify new sources of resistance or tolerance to abiotic stresses, such as drought. Indeed, the infection of a few RNA viruses delayed the onset of drought-related symptoms in different species [24, 55], underpinning the existence of molecular and physiological networks common to virus infection and plant resilience and, in turn, favoring the investigation of the viral molecular components that stimulate such beneficial effects. On this line, the 2b protein of CMV was found responsible for the improved performances of CMV-infected plants following drought [26], confirming that plant priming by a pathogen, or one of its components, activates metabolic pathways possibly leading to the maintenance of conditional outcomes [56]. The geminivirus TYLCV was also reported to promote drought tolerance in tomato and *N. benthamiana* under a reduced irrigation regime, and its C4 protein was suggested to contribute to increase the survival of Arabidopsis plants following water deprivation [33]. This prompted us to consider if and to what extent the C4 protein encoded by the related TYLCSV would confer a drought-tolerant phenotype to tomato, the natural host of this pathogen and a crop of extreme economic importance.

Indeed, TYLCSV C4 strongly delayed the dehydration effects of tomato plants, which perceived the stress later than WT controls. Notably, although C4–151 plants reached similar stomatal conductance levels typically associated to severe water deficit, they never attained the extremely low water potential values of WT controls. Moreover, we highlighted a faster physiological dynamic of recovery from drought in C4 compared to WT plants, strengthening the importance of this protein in enduring dehydration. Compared to WT, C4 plants did not mount the strong transcriptional reprogramming of ABA- and stress-responsive genes typical of severe drought conditions and experienced only a slight increase in ABA levels, thereby attesting a strongly attenuated stress perception. Accordingly, the morphometric analyses pointed out that the improved drought management of C4 plants could most likely depend on hydraulic signals (i.e. reduced basal transpiration rate in turn limiting excessive water loss, most likely due to the narrow xylem area) rather than biochemical (e.g. ABA) mechanisms.

Although more efforts are needed to further deepen the C4 mechanistic role, this work proves for the first time that the C4 protein of one of the two major TYLCD-inducing viruses of the Mediterranean region does affect physiological responses associated with water stress tolerance in tomato.

## Materials and methods

### Plant transformation

Tomato (*Lycopersicon esculentum* cv. Moneymaker) cotyledons were transformed with *Agrobacterium tumefaciens* C58 pGV2260 harboring plasmid pTOM102NT according to [56]. pTOM102NT is a derivative of pTOM100NT [57], having the TYLCSV C1 ORF start codon replaced by CTG and an in-frame TAA stop codon introduced 9 bases downstream, thus disabling the translation of Rep but not of the nested C4 ORF under the control of the CaMV E35S promoter. Transgene presence and expression were verified by PCR with primers END35S and TY2222(+) and by qRT-PCR with primers C4_72F and C4_179R, respectively (see Table S1 for primer list).

### Preliminary screening for drought tolerance of C4 expressing lines

T_3_ plants from three independent lines carrying the pTOM102NT cassette (lines C4–151, C4–153 and C4–156) and WT controls were grown in a glasshouse (average temperature 24.9 ± 5.35°C; relative humidity 42.3–61.8%). Each plant grew in a 1.5-L pot filled with a sandy-loam soil/expanded clay/peat mixture (3:2:4 by volume) substrate. Two months after sowing, a set of 4 plants per line and 4 WT plants were subjected to complete water withdrawal, while another set was watered every day. Plants were monitored daily for 7 days, measuring stem water potential (*Ψ_stem_*) and taking photographs when clear collapse occurred.

### Drought stress experimental outline

Three-month-old T_3_ plants of line C4–151 and WT plants (*n* = *14*) were grown as above, with additional control of maximal photosynthetic photon flux density (PPFD) (900–1200 μmol photons m^−2^ s^−1^). A photoperiod of 12-h-light/12-h-dark was obtained with halogen lamps to guarantee a minimum PPFD of 500–600 μmol photons m^−2^ s^−1^. Plants were either irrigated daily until water holding capacity (well-watered, WW; *n = 7*), or subjected to complete water withholding (water stressed, WS; *n = 7*). Plants were monitored daily for 14 days and re-watered to recover (recovered, REC) when they reached a severe water stress. Stomatal conductance (*g_s_*), transpiration (*E*) and net photosynthesis (*A_N_*) were monitored along the experiment. *Ψ_stem_* was measured at day 0 (WW plants), at day 5 and 8 (corresponding to maximum water stress for WT and C4–151 plants, respectively), and at day 14 (end of recovery). Samples for biochemical and molecular analyses were collected from WW and WS plants at the end of the drought stress treatment (day 5 and 8 for WT and C4–151 plants, respectively), as well as once recovery was completed (REC, day 14). A second experiment was repeated the year later, adopting the same experimental condition, to confirm the physiological trends.

### Relative water content analysis and leaf dehydration assay

Genotype-dependent changes in water loss were inspected gravimetrically monitoring the soil relative water content (RWC_soil_, %) on a separate set of potted WT and C4–151 plants, subjected to water deprivation. Besides, RWC_soil_ was also measured on pots containing a WT and a C4–151 plant, following the experimental approach proposed by [59].

Dynamics of leaf dehydration were evaluated on leaves detached from WT and C4–151 plants, either in WW and in WS conditions, monitoring gravimetrically the water loss over time (up to 450 minutes), and normalizing it on the leaf area. Leaf dehydration assays were carried out in a climatic chamber with constant T (25°C), RH (45%) and PPFD (200 μmol photons m^−2^ s^−1^), adopting the procedure detailed in [60].

### Stomatal phenotyping, leaf area, morphometric analysis and chlorophyll content index

Stomatal density, stomatal size and leaf area were measured on all plants at the beginning of the trial. Density (number of stomata mm^−2^) and size (mm^2^) of stomata from both abaxial and adaxial leaf surfaces were determined on scanning electron microscope (SEM) (TM3000, Hitachi High-Technologies Corp., Tokyo, Japan) images (120X magnification) on at least three randomly selected, non-overlapping images per leaf. Leaf area (cm^2^) was determined on pictures of apical, middle and basal full leaves of each plant. Images were processed with the ImageJ software (1.46r, NIH, https://imagej.nih.gov). Stomatal size was calculated as the product of length and width of 10 randomly selected stomata for each image [61].

At the beginning and end of the experiment, plant height, leaf number, stem diameter at the top, middle and basal level of all plants were measured. At the end of the experiment, root area and root biomass (dry weight) from WW and WS plants of both genotypes were measured. Root area was calculated by analyzing root images with the ImageJ software, while root biomass was determined by weighing samples after drying them at 70°C for 3–4 days. The Chlorophyll Content Index (CCI) was determined on the second and third fully developed leaves from the apex, using a portable chlorophyll meter SPAD 502 (CCM-200; Opti-Sciences), using two leaves per plant as biological replicates and taking three readings per leaf.

### Measurements of leaf gas exchanges and stem water potential

Stomatal conductance (*g_s_*), leaf transpiration (*E*) and net photosynthesis (*A_N_*) were monitored daily (between 9:00 and 12:00 h a.m.) on each plant using a portable infrared gas analyzer (LC*pro*-SD system, ADC BioScientific Ltd, Hertfordshire, UK) (6.25 cm^2^-leaf chamber; artificial irradiation 1200 μmol photon m^−2^ s^−1^; 25°C), measuring three random, fully expanded, non-senescing leaves exposed to direct sunlight. CO_2_ values were maintained at greenhouse conditions (400–450 ppm) for the whole experiment. Intrinsic water use efficiency (iWUE) was calculated as the *A_N_*/*g_s_* ratio.

*Ψ_stem_* was measured on equilibrated non-transpiring mature leaves, covered with aluminum foil and placed in a humidified plastic bag for at least 30 min before excision. After excision, leaves were allowed to equilibrate for >20 min in the dark, using a portable pressure chamber (1505D PMS Instrument Company, Albany, OR, USA).

### Analysis of hormone and proline contents and leaf osmolality

Hormone content was quantified according to [62], using 40 mg of lyophilized leaf tissue. For proline measurements, 50 mg of fresh leaf tissue were used, and osmolyte levels were quantified as reported by [63]. Leaf osmolality (Leaf_osm_) was determined starting from 30 mg of lyophilized leaf tissue following the method by [64]. Briefly, hot water extracts were carried out at 1:25 dilution (leaf DW/water volume, w/v) on ground samples in 1.5 mL microtubes. After shaking, the tubes were placed in a water bath at 90°C for 1 h. Cooled samples were centrifuged at 12′000 g for 5 min. Osmolality was then measured on 25 μl of supernatant using an osmometer (Micro-Digital Osmometer 7iM, Loser).

### Total RNA isolation and quantitative real-time PCR

Total RNA extracted from 100 mg of leaf sample with Trizol® (Thermo Fisher Scientific) was treated with TURBO DNase (Ambion). cDNA was synthetized from 1 μg total RNA using the High-Capacity cDNA reverse transcription kit (Applied Biosystems). qRT-PCR was conducted with the commercial iTaq Universal SYBR Green Supermix kit (Bio-Rad) in a CFX96 Real-Time PCR Detection system (Bio-Rad), using specific primers (Table S1). Relative transcript expression levels were normalized to the geometric mean of the Elongation factor (*SlEF*) and Ubiquitin (*SlUBI*) transcripts and calculated with the 2^−ΔCt^ method [65], using 3 biological replicates and 3 technical repetitions each for every sample and every condition.

### Morphological and structural analysis

Free-hand sections from plant stems collected between the 7^th^ and 8^th^ node from the apex were stained for 2 min with an aqueous solution of 50 mg ml^−1^ safranin, washed 3 times with water, and observed under a Leica DM 750 microscope equipped with a EC4 camera. Microscope images were then processed with the the ImageJ (1.46r, NIH, https://imagej.nih.gov) software to calculate vessel diameter, vessel density and the whole xylem cross-sectional area, using three stem sections per plant, and analyzing four plants per each genotype (*n = 12*).

### Statistical analysis

Significant differences among treatments and genotypes were analysed by a two-way analysis of variance (ANOVA). When ANOVA test indicated that either genotype (G: WT, C4–151) or treatment (T: WW, WS, REC) or their interaction (G × T) was significant, the Tukey’s honestly significant difference (HSD) post-hoc test was used to separate means (*P < 0.05*). The G main effects were statistically determined by a two-tailed Student’s *t*-test. The SPSS statistical software package (SPSS Inc., Cary, NC, USA, v.22) and the GraphPad Prism software (GraphPad Software, La Jolla, CA, USA v.6.01) were used to run the statistical analyses above reported and elaborate figure charts, respectively.

## Acknowledgements

The authors are grateful to Mario Tavazza (ENEA, Italy) for his support in generating transgenic plants and for fruitful discussions. We also thank Daniele Marian and Elena Zocca (IPSP-CNR) for technical assistance and plant care, and Stefano Pavarelli and Marco Pisani (INRIM, Italy) for technical support with SEM. This research was supported in part by the PRIMA2018_00090 Section 2 — GeMed Project and funded by the Italian Ministry of University and Research (MUR).

## Author contributions

E.N. and C.P. planned and designed the research. E.N., S.M., C.P., A.M., W.C., L.N., M.C., R.T., M.V. and F.S. performed experiments. E.N., S.M., C.P., A.M., W.C., M.C., R.T. and F.S. analyzed data. C.P. and E.N. wrote the manuscript. E.N., C.P., and S.M. reviewed and critically revised the manuscript. All authors read and approved the final manuscript.

## Data availability

All data can be found in the manuscript and in the Supporting Information.

## Conflict of interest

The authors have no conflicts of interest to declare.

## Supplementary data

Supplementary data is available at *Horticulture Research * online.

## Supplementary Material

Pagliarani_et_al_supplementary_material_R1_uhac164

## References

[1] Rosenzweig C, Elliott J, Deryng D et al. Assessing agricultural risks of climate change in the 21st century in a global gridded crop model intercomparison. *Proc Natl Acad Sci U S A*. 2014;111:3268–73. 24344314 10.1073/pnas.1222463110 PMC3948251

[2] Takahashi F, Kuromori T, Urano K et al. Drought stress responses and resistance in plants: from cellular responses to long-distance intercellular communication. *Front Plant Sci*. 2020;11:556972. 33013974 10.3389/fpls.2020.556972 PMC7511591

[3] Lovisolo C, Perrone I, Hartung W et al. An abscisic acid-related reduced transpiration promotes gradual embolism repair when grapevines are rehydrated after drought. *New Phytol*. 2008;180:642–51. 18700860 10.1111/j.1469-8137.2008.02592.x

[4] Kuromori T, Seo M, Shinozaki K. ABA transport and plant water stress responses. *Trends Plant Sci*. 2018;23:513–22. 29731225 10.1016/j.tplants.2018.04.001

[5] Claeys H, Inzé D. The agony of choice: how plants balance growth and survival under water-limiting conditions. *Plant Physiol*. 2013;162:1768–79. 23766368 10.1104/pp.113.220921 PMC3729759

[6] Hirayama T, Shinozaki K. Research on plant abiotic stress responses in the post-genome era: past, present and future. *Plant J*. 2010;61:1041–52. 20409277 10.1111/j.1365-313X.2010.04124.x

[7] Zhou R, Kong L, Wu Z et al. Physiological response of tomatoes at drought, heat and their combination followed by recovery. *Physiol Plant*. 2019;165:144–54. 29774556 10.1111/ppl.12764

[8] Panno S, Davino S, Caruso AG et al. A review of the most common and economically important diseases that undermine the cultivation of tomato crop in the Mediterranean basin. *Agronomy*. 2021;11:2188.

[9] Chattopadhyay C, Birah A, Jalali BL. Climate change: impact on biotic stresses afflicting crop plants. In: Peshin R, Dhawan A, eds. Natural Resource Management: Ecological Perspectives. Sustainability in Plant and Crop Protection. Springer: Charm, 2019,133–46.

[10] Rahman A, Sinha KV, Sopory SK et al. Influence of virus-host interactions on plant response to abiotic stress. *Plant Cell Rep*. 2021;40:2225–45. 34050797 10.1007/s00299-021-02718-0

[11] Prasad A, Sett S, Prasad M. Plant-virus-abiotic stress interactions: a complex interplay. *Environ Exp Bot*. 2022;199:104869.

[12] Yan Z, Wolters AA, Navas-Castillo J et al. The global dimension of tomato yellow leaf curl disease: current status and breeding perspectives. *Microorganisms*. 2021;9:740. 33916319 10.3390/microorganisms9040740 PMC8066563

[13] Kheyr-Pour A, Bendahmane M, Matzeit V et al. Tomato yellow leaf curl virus from Sardinia is a whitefly-transmitted monopartite geminivirus. *Nucl Acids Res*. 1991;19:6763–9. 1840676 10.1093/nar/19.24.6763 PMC329307

[14] Medina-Puche L, Orìlio AF, Zerbini FM et al. Small but mighty: functional landscape of the versatile geminivirus encoded C4 protein. *PLoS Pathog*. 2021;17:e1009915. 34618877 10.1371/journal.ppat.1009915 PMC8496806

[15] De Jupin I, Kouchkovsky F, Jouanneau F et al. Movement of tomato yellow leaf curl geminivirus (TYLCV): involvement of the protein encoded by ORF C4. *Virology*. 1994;204:82–90. 8091687 10.1006/viro.1994.1512

[16] Rojas MR, Jiang H, Salati R et al. Functional analysis of proteins involved in movement of the monopartite begomovirus, tomato yellow leaf curl virus. *Virology*. 2001;291:110–25. 11878881 10.1006/viro.2001.1194

[17] Luna AP, Morilla G, Voinnet O et al. Functional analysis of gene-silencing suppressors from tomato yellow leaf curl disease viruses. *Mol Plant-Microbe Interact*. 2012;25:1294–306. 22712505 10.1094/MPMI-04-12-0094-R

[18] Rosas-Diaz T, Zhang D, Fan P et al. A virus-targeted plant receptor-like kinase promotes cell-to-cell spread of RNAi. *Proc Natl Acad Sci U S A*. 2018;115:1388–93. 29363594 10.1073/pnas.1715556115 PMC5819414

[19] Rigden JE, Krake LR, Rezaian MA et al. ORF C4 of tomato leaf curl geminivirus is a determinant of symptom severity. *Virology*. 1994;204:847–50. 7941358 10.1006/viro.1994.1606

[20] Mei Y, Ma Z, Wang Y et al. Geminivirus C4 antagonizes the HIR-mediated hypersensitive response by inhibiting the HIR1 self-interaction and promoting degradation of the protein. *New Phytol*. 2020;225:1311–26. 31537050 10.1111/nph.16208

[21] Jing C, Li P, Zhang J et al. The Malvastrum yellow vein virus C4 protein promotes disease symptom development and enhances virus accumulation in plants. *Front Microbiol*. 2019;10:2425. 31708897 10.3389/fmicb.2019.02425 PMC6823909

[22] Aguilar E, Cutrona C, del Toro FJ et al. Virulence determines beneficial trade-offs in the response of virus-infected plants to drought via induction of salicylic acid. *Plant Cell Environ*. 2017;40:2909–30. 28718885 10.1111/pce.13028

[23] Carr JP . Exploring how viruses enhance plants' resilience to drought and the limits to this form of viral payback. *Plant Cell Environ*. 2017;40:2906–8. 28898417 10.1111/pce.13068

[24] Xu P, Chen F, Mannas JP et al. Virus infection improves drought tolerance. *New Phytol*. 2008;180:911–21. 18823313 10.1111/j.1469-8137.2008.02627.x

[25] Bostock RM, Pye MF, Roubtsova TV. Predisposition in plant disease: exploiting the nexus in abiotic and biotic stress perception and response. *Annu Rev Phytopathol*. 2014;52:517–49. 25001451 10.1146/annurev-phyto-081211-172902

[26] Westwood JH, Mccann L, Naish M et al. A viral RNA silencing suppressor interferes with abscisic acid-mediated signalling and induces drought tolerance in *Arabidopsis thaliana*. *Mol Plant Pathol*. 2013;14:158–70. 23083401 10.1111/j.1364-3703.2012.00840.x PMC6638696

[27] Pantaleo V, Vitali M, Boccacci P et al. Novel functional microRNAs from virus-free and infected Vitis vinifera plants under water stress. *Sci Rep*. 2016;6:20167. 26833264 10.1038/srep20167 PMC4735847

[28] Ascencio-Ibáñez JT, Sozzani R, Lee TJ et al. Global analysis of Arabidopsis gene expression uncovers a complex array of changes impacting pathogen response and cell cycle during geminivirus infection. *Plant Physiol*. 2008;148:436–54. 18650403 10.1104/pp.108.121038 PMC2528102

[29] Hanley-Bowdoin L, Bejarano ER, Robertson D et al. Geminiviruses: masters at redirecting and reprogramming plant processes. *Nat Rev Microbiol*. 2013;11:777–88. 24100361 10.1038/nrmicro3117

[30] Chen T, Lv Y, Zhao T et al. Comparative transcriptome profiling of a resistant vs. susceptible tomato (*Solanum lycopersicum*) cultivar in response to infection by tomato yellow leaf curl virus. *PLoS One*. 2013;8:e80816. 24260487 10.1371/journal.pone.0080816 PMC3832472

[31] Miozzi L, Napoli C, Sardo L et al. Transcriptomics of the interaction between the monopartite phloem-limited geminivirus tomato yellow leaf curl Sardinia virus and *Solanum lycopersicum* highlights a role for plant hormones, autophagy and plant immune system fine tuning during infection. *PLoS One*. 2014;9:e89951. 24587146 10.1371/journal.pone.0089951 PMC3938563

[32] Anfoka G, Moshe A, Fridman L et al. Tomato yellow leaf curl virus infection mitigates the heat stress response of plants grown at high temperatures. *Sci Rep*. 2016;6:19715. 26792235 10.1038/srep19715 PMC4726131

[33] Corrales-Gutierrez M, Medina-Puche L, Yu Y et al. The C4 protein from the geminivirus tomato yellow leaf curl virus confers drought tolerance in Arabidopsis through an ABA-independent mechanism. *Plant Biotech J*. 2019;18:1121–3. 10.1111/pbi.13280 PMC7152601 31637850

[34] Shteinberg M, Mishra R, Anfoka G et al. Tomato yellow leaf curl virus (TYLCV) promotes plant tolerance to drought. *Cell*. 2021;10:2875. 10.3390/cells10112875 PMC8616339 34831098

[35] Mishra R, Shteinberg M, Shkolnik D et al. Interplay between abiotic (drought) and biotic (virus) stresses in tomato plants. *Mol Plant Pathol*. 2022;23:475–88. 34970822 10.1111/mpp.13172 PMC8916204

[36] Chitarra W, Pagliarani C, Maserti B et al. Insights on the impact of arbuscular mycorrhizal symbiosis on tomato tolerance to water stress. *Plant Physiol*. 2016;171:1009–23. 27208301 10.1104/pp.16.00307 PMC4902612

[37] Roshan P, Kulshreshtha A, Hallan V. Identification of host cellular targets of AC4 and AV2 proteins of tomato leaf curl Palampur virus and their sub-cellular localization studies. *Virus Disease*. 2017;28:390–400. 29291230 10.1007/s13337-017-0405-5 PMC5747847

[38] Raghavendra AS, Gonugunta VK, Christmann A et al. ABA perception and signalling. *Trends Plant Sci*. 2010;15:395–401. 20493758 10.1016/j.tplants.2010.04.006

[39] Visentin I, Pagliarani C, Deva E et al. A novel strigolactone-miR156 module controls stomatal behaviour during drought recovery. *Plant Cell Environ*. 2020;43:1613–24. 32196123 10.1111/pce.13758

[40] Sacco A, Greco B, Di Matteo A et al. Evaluation of tomato genetic resources for response to water deficit. *Am J Plant Sci*. 2013;04:131–45.

[41] Yoshiba Y, Kiyosue T, Nakashima K et al. Regulation of levels of proline as an osmolyte in plants under water stress. *Plant Cell Physiol*. 1997;38:1095–102. 9399433 10.1093/oxfordjournals.pcp.a029093

[42] Hu C, Delauney AJ, Verma D. A bifunctional enzyme (delta 1-pyrroline-5- carboxylate synthetase) catalyzes the first two steps in proline biosynthesis in plants. *Proc Natl Acad Sci U S A*. 1992;89:9354–8. 1384052 10.1073/pnas.89.19.9354 PMC50125

[43] Kiyosue T, Yoshiba Y, Yamaguchi-Shinozaki K et al. A nuclear gene encoding mitochondrial proline dehydrogenase, an enzyme involved in proline metabolism, is upregulated by proline but downregulated by dehydration in Arabidopsis. *Plant Cell*. 1996;8:1323–35. 8776899 10.1105/tpc.8.8.1323 PMC161248

[44] Bertolino LT, Caine RS, Gray JE. Impact of Stomatal density and morphology on water-use efficiency in a changing world. *Front Plant Sci*. 2019;10:225. 30894867 10.3389/fpls.2019.00225 PMC6414756

[45] Fan P, Aguilar E, Bradai M et al. The receptor-like kinases BAM1 and BAM2 are required for root xylem patterning. *Proc Natl Acad Sci U S A*. 2021;118:e2022547118. 33723062 10.1073/pnas.2022547118 PMC7999944

[46] López R, Cano FJ, Martin-StPaul NK et al. Coordination of stem and leaf traits define different strategies to regulate water loss and tolerance ranges to aridity. *New Phytol*. 2021;230:497–509. 33452823 10.1111/nph.17185

[47] Lovisolo C, Schubert A. Effects of water stress on vessel size and xylem hydraulic conductivity in Vitis vinifera L. *J Exp Bot*. 1998;49:693–700.

[48] Zouine M, Fu Y, Chateigner-Boutin AL et al. Characterization of the tomato ARF gene family uncovers a multi-levels post-transcriptional regulation including alternative splicing. *PLoS One*. 2014;9:e84203. 24427281 10.1371/journal.pone.0084203 PMC3888382

[49] Liu S, Zhang Y, Feng Q et al. Tomato AUXIN RESPONSE FACTOR 5 regulates fruit set and development via the mediation of auxin and gibberellin signaling. *Sci Rep*. 2018;8:2971. 29445121 10.1038/s41598-018-21315-y PMC5813154

[50] Ghosh D, Chakraborty S. Molecular interplay between phytohormones and geminiviruses: a saga of a never-ending arms race. *J Exp Bot*. 2021;72:2903–17. 33577676 10.1093/jxb/erab061

[51] Wu M, Ding X, Fu X et al. Transcriptional reprogramming caused by the geminivirus tomato yellow leaf curl virus in local or systemic infections in *Nicotiana benthamiana*. *BMC Genomics*. 2019;20:542. 31272383 10.1186/s12864-019-5842-7 PMC6611054

[52] Vinutha T, Vanchinathan S, Bansal N et al. Tomato auxin biosynthesis/signaling is reprogrammed by the geminivirus to enhance its pathogenicity. *Planta*. 2020;252:51. 32940767 10.1007/s00425-020-03452-9

[53] Bouzroud S, Gouiaa S, Hu N et al. Auxin response factors (ARFs) are potential mediators of auxin action in tomato response to biotic and abiotic stress (*Solanum lycopersicum*). *PLoS One*. 2018;13:e0193517. 29489914 10.1371/journal.pone.0193517 PMC5831009

[54] Fraile A, García-Arenal F. The coevolution of plants and viruses: resistance and pathogenicity. *Natural and Engineered Resistance to Plant Viruses, Part II*. 2010;76:1–32. 10.1016/S0065-3527(10)76001-2 20965070

[55] Dastogeer K . Influence of fungal endophytes on plant physiology is more pronounced under stress than well-watered conditions: a meta-analysis. *Planta*. 2018;248:1403–16. 30121874 10.1007/s00425-018-2982-y

[56] Conrath U, Pieterse CMJ, Mauch-Mani B. Priming in plant pathogen interactions. *Trends Plant Sci*. 2002;7:210–6. 11992826 10.1016/s1360-1385(02)02244-6

[57] Brunetti A, Tavazza M, Noris E et al. High expression of truncated viral rep protein confers resistance to tomato yellow leaf curl virus in transgenic tomato plants. *Mol Plant-Microbe Interact*. 1997;10:571–9.

[58] Noris E, Accotto GP, Tavazza R et al. Resistance to tomato yellow leaf curl geminivirus in *Nicotiana benthamiana* plants transformed with a truncated viral C1 gene. *Virology*. 1996;224:130–8. 8862407 10.1006/viro.1996.0514

[59] Manacorda CA, Gudesblat G, Sutka M et al. TuMV triggers stomatal closure but reduces drought tolerance in Arabidopsis. *Plant Cell Environ*. 2021;44:1399–416. 33554358 10.1111/pce.14024

[60] Hopper DW, Ghan R, Cramer GR. A rapid dehydration leaf assay reveals stomatal response differences in grapevine genotypes. *Hortic Res*. 2014;1:2. 26504528 10.1038/hortres.2014.2 PMC4591676

[61] Kardiman R, Ræbild R. Relationship between stomatal density, size and speed of opening in Sumatran rainforest species. *Tree Physiol*. 2018;38:696–705. 29186586 10.1093/treephys/tpx149

[62] Pagliarani C, Gambino G, Ferrandino A et al. Molecular memory of Flavescence dorée phytoplasma in recovering grapevines. *Hortic Res*. 2020;7:126. 32821409 10.1038/s41438-020-00348-3 PMC7395728

[63] Mannino G, Nerva L, Gritli T et al. Effects of different microbial inocula on tomato tolerance to water deficit. *Agronomy*. 2020;10:170.

[64] Callister AN, Arndt SK, Adams MA. Comparison of four methods for measuring osmotic potential of tree leaves. *Physiol Plant*. 2006;127:383–92.

[65] Livak KJ, Schmittgen TD. Analysis of relative gene expression data using real-time quantitative PCR and the 2−ΔΔCT method. *Methods*. 2001;25:402–8. 11846609 10.1006/meth.2001.1262

