# Supplementary material for: The C4 protein of tomato yellow leaf curl Sardinia virus primes drought tolerance in tomato through morphological adjustments
Source: Hortic Res. 2022 Jul 27;9:uhac164. doi: 10.1093/hr/uhac164 (PMC9613725; doi:10.1093/hr/uhac164)

Supplementary Information

The C4 protein of tomato yellow leaf curl Sardinia virus primes drought tolerance in tomato through morphological adjustments

Chiara Pagliarani,[1] Amedeo Moine,[1] Walter Chitarra,[1,2] Luca Nerva,[1,2] Marco Catoni,[1,3] Raffaela Tavazza,[4] Slavica Matić,[1,*] Marta Vallino[1], Francesca Secchi[5] and Emanuela Noris[1,*]

Supplementary Table S1. List of oligonucleotides used for qRT-PCR experiments.

Primer name	Primer sequence	Target gene (Gene description and Solyc code)	Reference
LeNCED1_F	ACCCACGAGTCCAGATTTC	9-cis-epoxycarotenoid	66
LeNCED1_R	GGTTCAAAAAGAGGGTTAGC		
LeTas14_F	CAATACGGCAATCAAGACCAAA	Dehydrin TAS14 Solyc02g084850.2.1	36
LeTas14_R	TGTTTCTTGGACATGGTTTCCA		
LePIP1;1_F	GTGTCTCGGTGCCATCTGTG	Tomato aquaporin Solyc08g008050.2	36
LePIP1;1_R	ATCCGGGTTGAACCACATTG		
SlCYP707A1_F	CCCAGAGTTCTTTCCTGATCCACAA	ABA-8'-hidroxylase Solyc04g078900.3	67
SlCYP707A1_R	GAATGCCACTACCAGATCCTACCAC		
SlCYP707A2_F	TCGAAAAAGGATACAATTCGATGCC	ABA-8'-hidroxylase Solyc08g075320.3	67
SlCYP707A2_R	CTGCAATTTGTTCGTCAGTGAGTCC		
SlP5CS1_F	TCCTCCCCGTGATCCAGATT	delta1-pyrroline-5-carboxylate synthetase 1 Solyc08g043170.2	This study
SlP5CS1_R	AGTCATGCCTCCTCTTCCCA		
SlPDH_F	TGATGCGGAAGACACAACCA	proline dehydrogenase Solyc02g089620.2.1	This study
SlPDH_R	ATTCGTTCCTTGGCGTCCTT		
SlARF5_F	CCTTCAGAGTTTGTCATTCCT	Auxin response factor 5 Solyc04g081240.2.1	48
SlARF5_R	AACATCATTCCAAATCTCATACC		
SlARF8_F	TGGGAAAGGAAGAGGCTGAA	Auxin response factor 8 Solyc02g037530.2.1	52
SlARF8_R	GCGATCCAAGAGATGGCATT		
SlIAA9_F	TCCAGCATTTCTAGTAGGCGA	Auxin-responsive protein IAA9 Solyc04g076850.2.1	This study
SlIAA9_R	TGCTGAGCTATCCAAGACCA		
SlIAA4_F	GGTTTACCAGGGAGGACAG	Auxin-responsive protein IAA4 Solyc06g053840.2.1	49
SlIAA4_R	GGGATCTGAAATGGAGTTTG		
SlIAA14_F	TGGCCACCAGTGAGATCATT	Auxin-responsive protein IAA14 Solyc09g083290.2.1	This study
SlIAA14_R	CACCATCCATGCAAACTTTGAC		
LeEF_F	CTCCATTGGGTCGTTTTGCT	Elongation Factor	68
LeEF_R	GGTCACCTTGGCACCAGTTG		
LeUbi_F	CTTGTTGGGGTAATCCTCAG	Ubiquitin	69
LeUbi_R	CTTGTTGGGGTAATCCTCAG		
C4_72F	TTCTTCGACCTGGTATCCCC	TYLCSV C4	This study
C4_179R	AACATCTCCGTCCTTGTCGA		
END-35S	TTCGCAAGACCCTTCCTCTA	TYLCSV C4	70
TY2222(+)	GTCGTTGGCTGTCTGTTGTC		

References not included in the reference list of the main text:

66 López-Ráez, J.A. et al. Does abscisic acid affect strigolactone biosynthesis? *New Phytol.* **187,** 343–354 (2010).

67 Sun, L. et al. Suppression of 9-cis-*Epoxycarotenoid dioxygenase*, which encodes a key enzyme in abscisic acid biosynthesis, alters fruit texture in transgenic tomato. *Plant Physiol.* **158**, 283–298 (2012).

68 Digilio, M.C. et al. Molecular and chemical mechanisms involved in aphid resistance in cultivated tomato. *New Phytol.* **187**, 1089–1101 (2010).

69 Weiss, J. & Egea-Cortines, M. Transcriptomic analysis of cold response in tomato fruits identifies dehydrin as a marker of cold stress. *J. Appl. Genet.* **50**, 311–319 (2009).

70 Davino, S., Davino, M. & Accotto, G.P. A single-tube PCR assay for detecting viruses and their recombinants that cause tomato yellow leaf curl disease in the Mediterranean basin. *J. Virol. Methods* **147**: 93–98 (2008).

Supplementary Fig. S1. Particular of leaf morphology defects in C4 transgenic lines (C4-151, C4-153 and C4-156) in comparison with wild type (WT). Images were taken from plants maintained in well-watered conditions. The bar in each image is 2 cm.

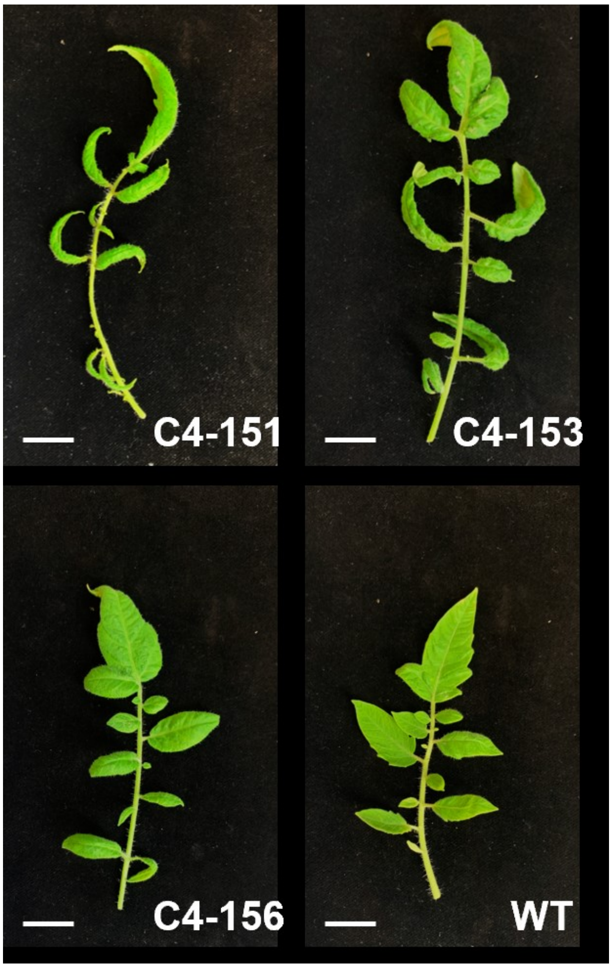

Supplementary Fig. S2. Effects of C4 expression on tomato physiological performances during a drought and recovery time-course (II experimental trial). Dynamic changes in the rates of A) stomatal conductance (g_s, mmol H_2O m^{-2} s^{-1}), B) assimilation (A_N, µmol CO_2 m^{-2} s^{-1}), C) transpiration (E, mmol H_2O m^{-2} s^{-1}), and (D) iWUE (µmol CO_2 $mmol^{-1}$ H_2O) of WT (Moneymaker, triangles) and C4-151 plants (circles) under well-watered (WW, black-filled symbols) and water stress conditions followed by recovery (WS/REC, empty symbols). Water withdrawal started at Day 1, while recovery started following irrigation at Days 7 and 11 for WT and C4-151 plants, respectively (black arrows). (D) Stem water potential (Ψ_{stem}) of WT and C4-151 plants measured under different water conditions (WW = Day 1; WS = Day 7 and Day 11; and REC = Day 15 of the time-course reported in A to C panel). In panels (A-D), lower case letters indicate significant differences among the four conditions within each experimental day, as assessed by Tukey's *HSD* test ($p \leq 0.05$), while the red asterisk, when present, denotes significant differences between WT and C4-151 tomatoes at the end of the WS treatment (ie. day 7 *vs* day 11, as highlighted by the red square) as determined by a two-tailed Student's *t* test (*$p \leq 0.05$). In D), the asterisks denote significant differences between genotypes under the same condition, as determined by a two-tailed Student's *t* test (***$p \leq 0.001$). Data are the mean ± SE (n = 7).

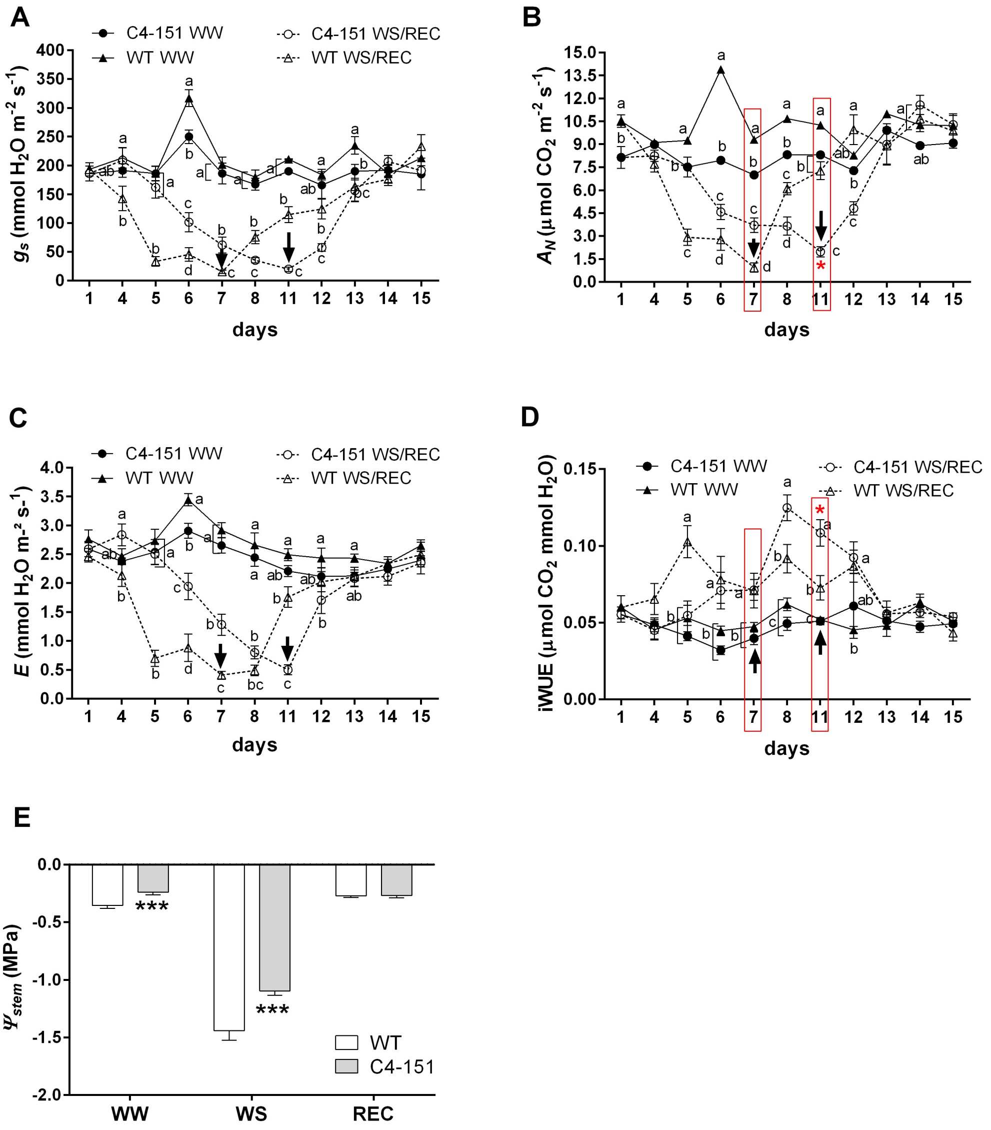

Supplementary Fig. S3. Differences in the susceptibility of WT and C4-151 plants to water stress as revealed by a two plants per pot experiment. A-B) Representative images taken on A) fully watered plants at the beginning of the experiment, and B) plants at the end of the water stress time-course. C) Changes in the soil relative water content (RWC_{soil}, %) gravimetrically monitored over the duration of the WS time-course on the pots containing WT and C4-151 plants (two plants per pot experiment). D) Stem water potential (Ψ_{stem}, MPa) of WT and C4-151 plants grown in the same pot and subjected to different water conditions: WW = well-watered, WS = water stressed. Ψ_{stem} was measured on WW and WS plants at the end of the experimental trial. The asterisks, when present, denote significant differences between genotypes under the same condition, as determined by a two-tailed Student's *t* test (***$p \leq 0.001$). Data are the mean ± SE (n = 6).

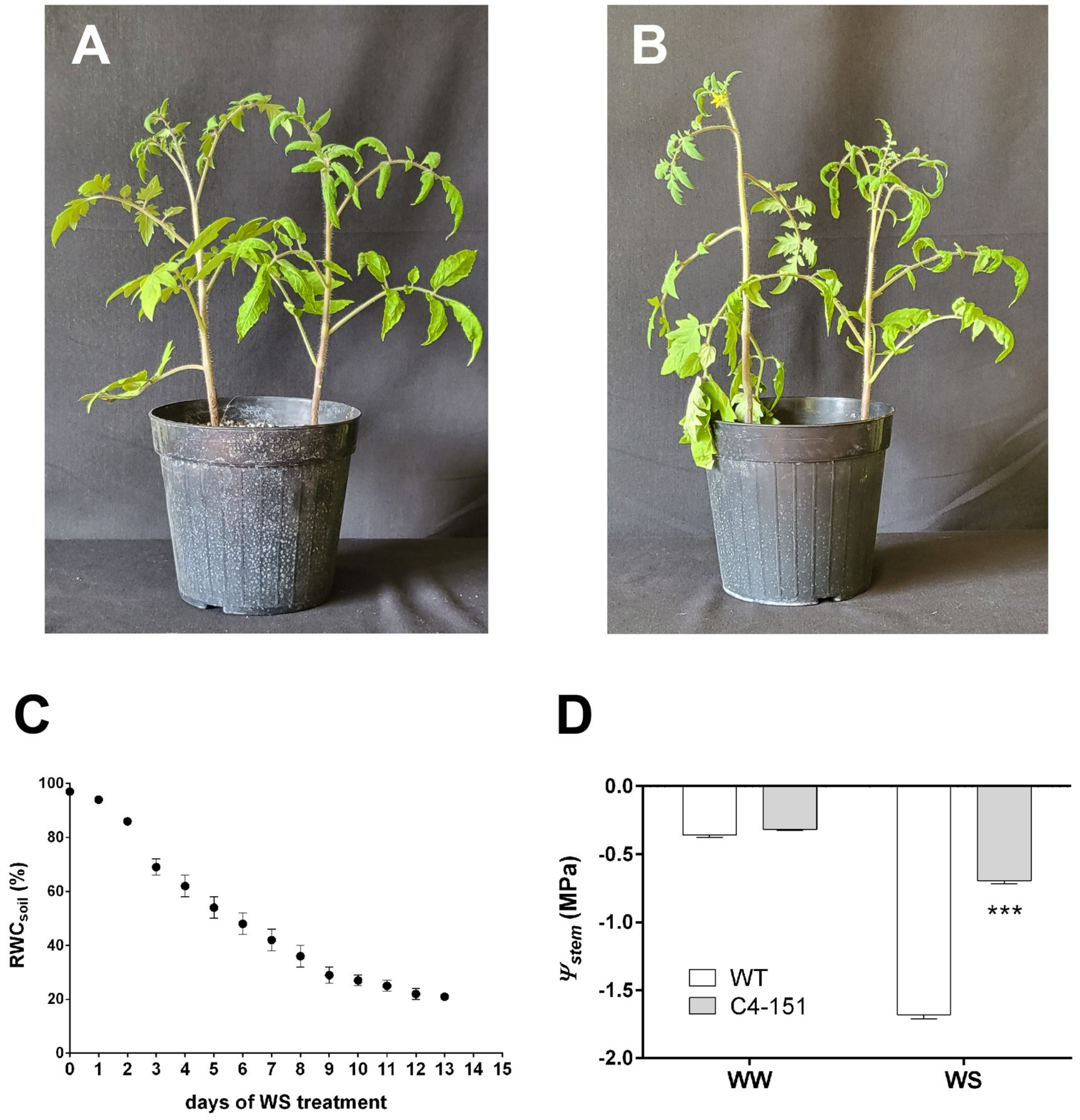

Supplementary Fig. S4. Measurement of chlorophyll content index (CCI, SPAD units) in WT and C4-151 plants. Data in (A, B) refer to the first experimental trial, while data in (C, D) refer to the second experiment. Data were taken at the beginning (A, C) and end (B, D) of the water stress experiment. Data represent the mean ± SE (n = 14 and n = 7 in (A, C) and (B, D), respectively). In (A) and (C), when present, the asterisks denote significant difference between genotypes, as assessed by a two-tailed Student's *t* test (** $p \leq 0.01$). In (B) and (D), the significance of genotype, treatment, and genotype × treatment (G × T) interaction was assessed by Tukey's *HSD* test and the corresponding results are given above each graph in the figure panel; $P \leq 0.01$ (**); n.s. = not significant. Lower case letters above bars are reported when the G × T interaction and/or Genotype (G) main effects are statistically significant as attested by Tukey's *HSD* or Student's *t* test, respectively. WW = well-watered; WS = water-stressed.

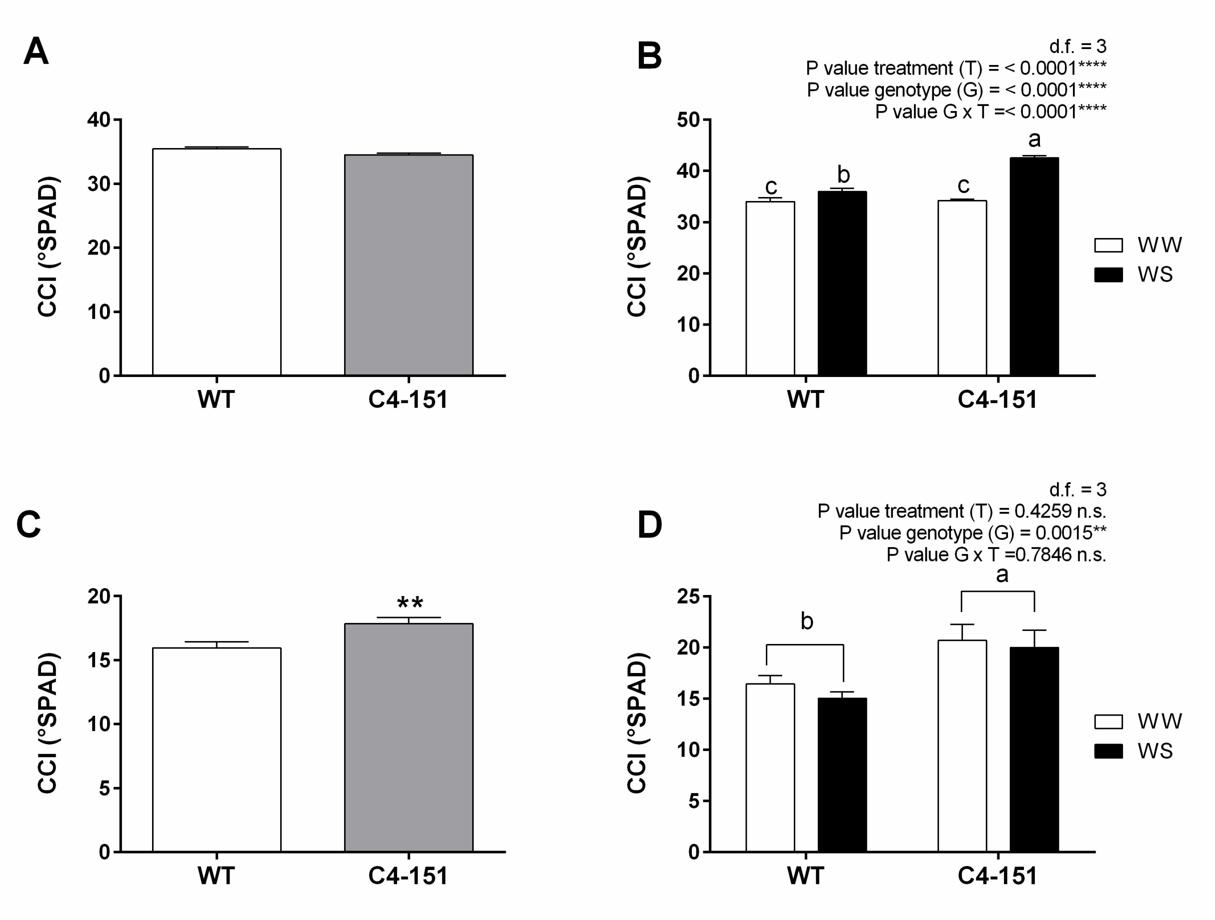

Supplementary Fig. S5. Biometric measurements of WT and C4-151 plants (I experimental trial). Data were collected at the beginning (A, C, E) and end (B, D, F) of the water stress experiment. Data represent the mean ± SE (n=14 and n=7 in A, C, E and B, D, F respectively). In (A, C, E) asterisks, when present, denote significant differences attested by a two-tailed Student's *t* test (*$p \leq 0.05$; ** $p \leq 0.01$; *** $p \leq 0.001$). In B, D, F the significance of genotype (G), treatment (T), and genotype × treatment (G x T) interaction was assessed by Tukey's *HSD* test and the corresponding results are given above each graph in the figure panel (for B and D) or at the end of the figure caption (for F); $p \leq 0.05$ (*); $p \leq 0.01$ (**); $p \leq 0.0001$ (****); n.s.=not significant. Lower case letters are reported when the G × T interaction and/or Genotype (G) main effects are statistically significant, as attested by Tukey's *HSD* or Student's *t* test, respectively. Stem diameter: Top - p value (T) = < 0.0001****; p value (G) = < 0.0001****; p value G x T = 0.0094**; Middle - p value (T) = 0.0103*; P value (G) = < 0.0001****; p value G x T = 0.6526 n.s.; Bottom - p value (T) = 0.0872 n.s.; p value (G) = 0.0187*; p value G x T = 0.0778 n.s. WW = well-watered; WS = water-stressed.

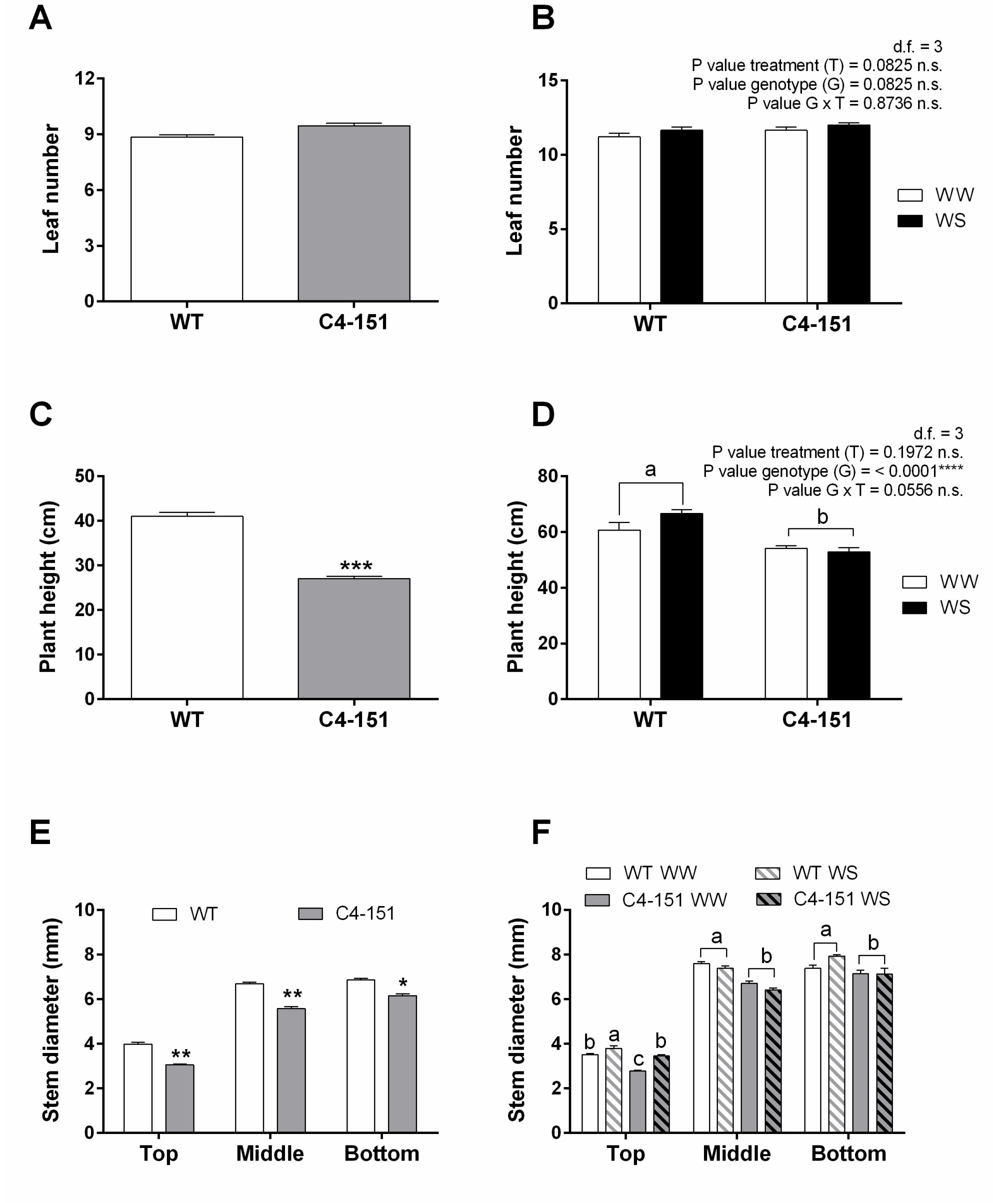

Supplementary Fig. S6. Biometric measurements of WT and C4-151 plants (II experimental trial). Data were collected at the beginning (A, C, E) and end (B, D, F) of the water stress experiment. Data represent the mean ± SE (n = 14 and n = 7 in A, C, E and B, D, F respectively). In (A, C, E) asterisks, when present, denote significant differences attested by a two-tailed Student's *t* test (***$p \leq 0.001$). In (B, D, F) the significance of genotype (G), treatment (T), and genotype × treatment (G × T) interaction was assessed by Tukey's *HSD* test and the corresponding results are given above each graph in the figure panel (for B and D) or at the end of the figure caption (for F); $p \leq 0.05$ (*); $p \leq 0.01$ (**); n.s. = not significant. Lower case letters are reported when the G × T interaction and/or Genotype (G) main effects are statistically significant, as attested by Tukey's *HSD* or Student's *t* test, respectively. Stem diameter: Top - p value (T) = 0.3697 n.s.; p value (G) = 0.1734 n.s.; p value G x T = 0.0165*; Middle - p value (T) = 0.0888 n.s.; p value (G) = 0.0124*; p value G x T = 0.9137 n.s.; Bottom - p value (T) = 0.1957 n.s.; p value (G) = 0.7280 n.s.; p value G x T = 0.1388 n.s. WW = well-watered; WS = water-stressed.

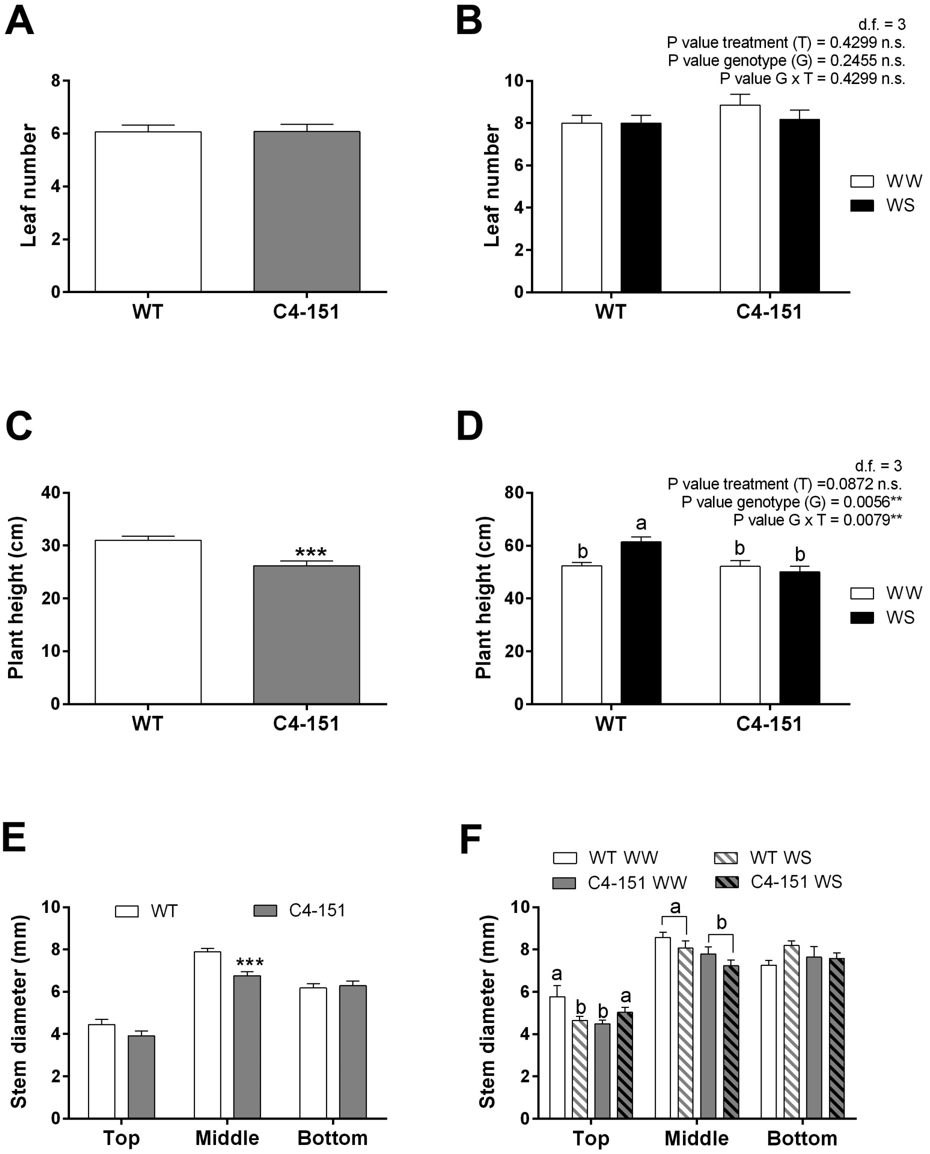

Supplementary Fig. S7. Root morphology of WT and C4-151 plants. Representative images of roots from (A) WT and (B) C4-151 tomato plants; magnification bar: 10 cm. Analysis of (C) root area and (D) root biomass of WT and C4-151 plants in well-watered (WW) and water stressed (WS) conditions; data represent the mean ± SE (n = 4). The significance of genotype (G), treatment (T), and genotype × treatment (G × T) interaction was assessed by Tukey's *HSD* test and the corresponding results are given above each graph in the figure panel; $p \leq 0.001$ (***), $p \leq 0.0001$ (****); n.s. = not significant. Lower case letters are reported when the G × T interaction and/or Genotype (G) main effects are statistically significant, as attested by Tukey's *HSD* or Student's t test, respectively.

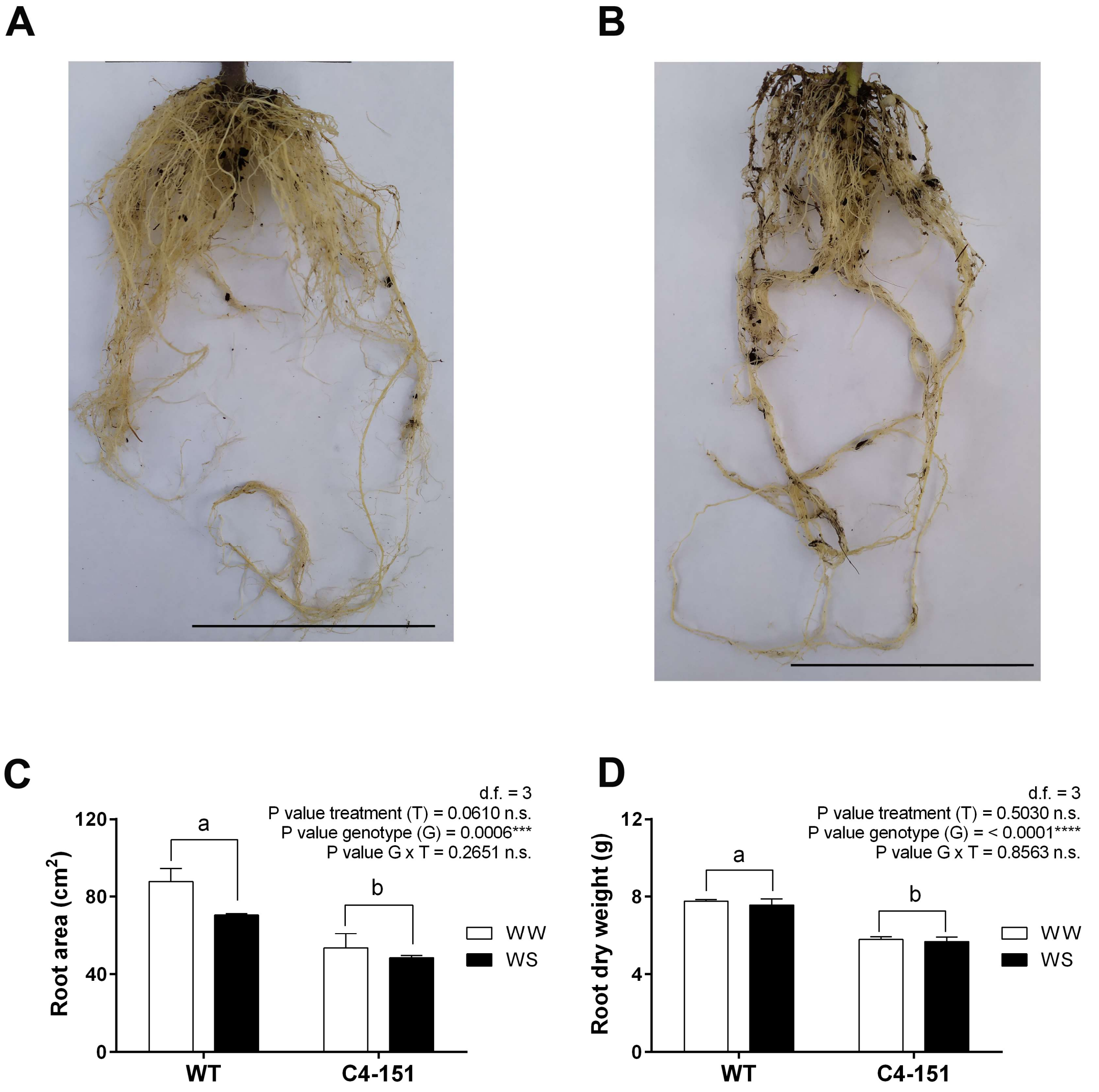

Supplementary Fig. S8. Morphological observations of stomata from the adaxial leaf surface. Representative images of stomata from (A) WT and (B) C4-151 tomato plants obtained by Scanning Electron Microscope (SEM) observations (150x magnification) of intact leaves (adaxial leaf surface) of the two genotypes. The inset in each image shows a magnified detail of stomata. Data of (C) stomatal density and (D) stomatal area of WT and C4-151 plants represent the mean ± SE (*n = 21* and *n = 210* for C and D respectively). When present, the asterisks denote significant difference between genotypes, as assessed by a two-tailed Student's *t* test (***p ≤ 0.01*; ****p ≤ 0.001*).

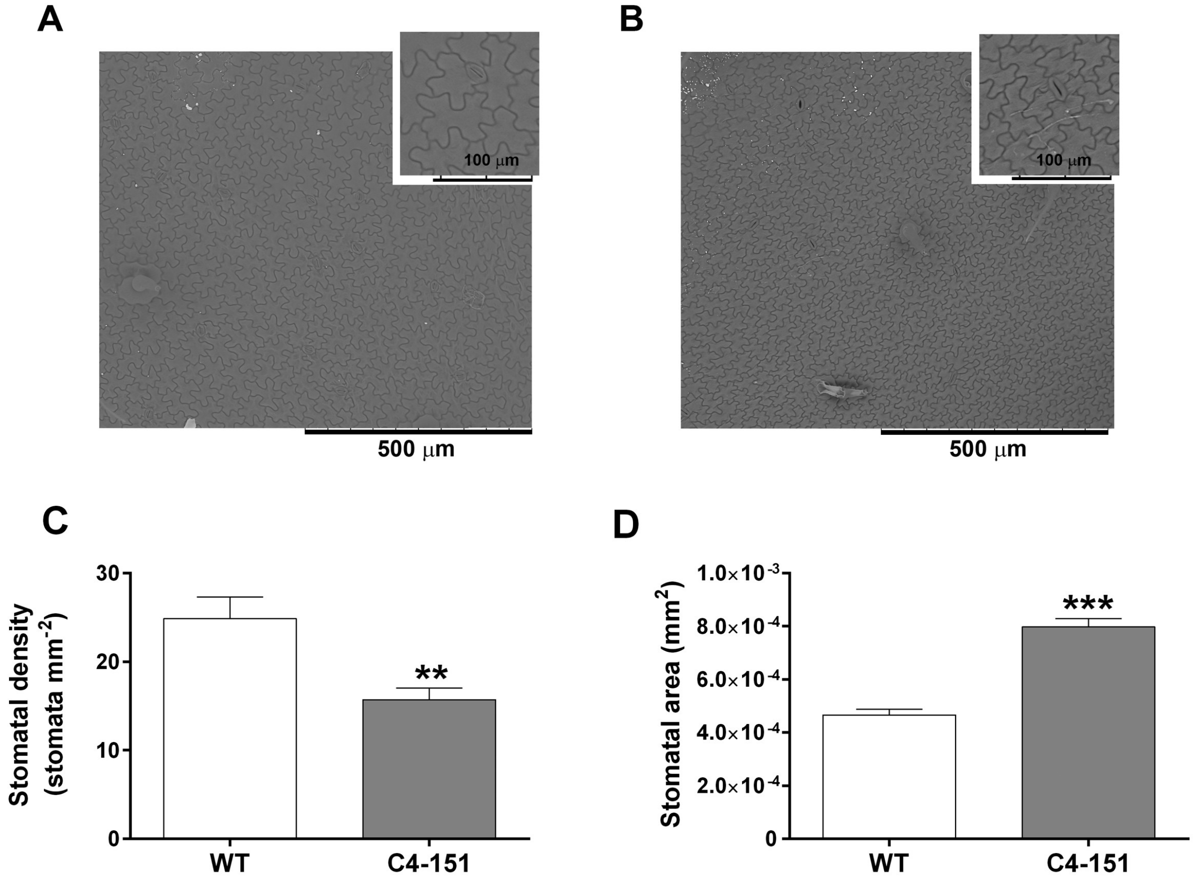

Supplementary Fig. S9. Focus on auxin metabolism. Content of indolacetic acid (IAA) and expression profiles of key genes positively (*SlIARF5*, *SlARF8*) or negatively regulating (*SlIAA4*, *SlIAA9*, *SlIAA14*) the auxin signaling pathway in leaf samples from WT and C4-151 tomato plants subjected to water stress treatment (WS) and recovery (REC), in comparison with well-watered controls (WW). Ubiquitin (*SlUBI*) and Elongation factor 1α (*SlEF*) genes were both used as endogenous housekeeping controls for the normalization of transcript levels. Significance of genotype, treatment, and genotype × treatment (G × T) interaction was assessed by Tukey's *HSD* test for $p \leq 0.05$ (*), $p \leq 0.01$ (**), $p \leq 0.001$ (***), and $p \leq 0.0001$ (****) and the corresponding results are given above each graph in the figure panel; n.s. = not significant. Lower case letters above bars are reported when the G × T interaction and/or genotype (G) main effects are statistically significant as attested by Tukey's *HSD* or Student's *t*-test, respectively. Error bars represent SE. Three independent biological replicates with three technical replicates each were used for the analysis.

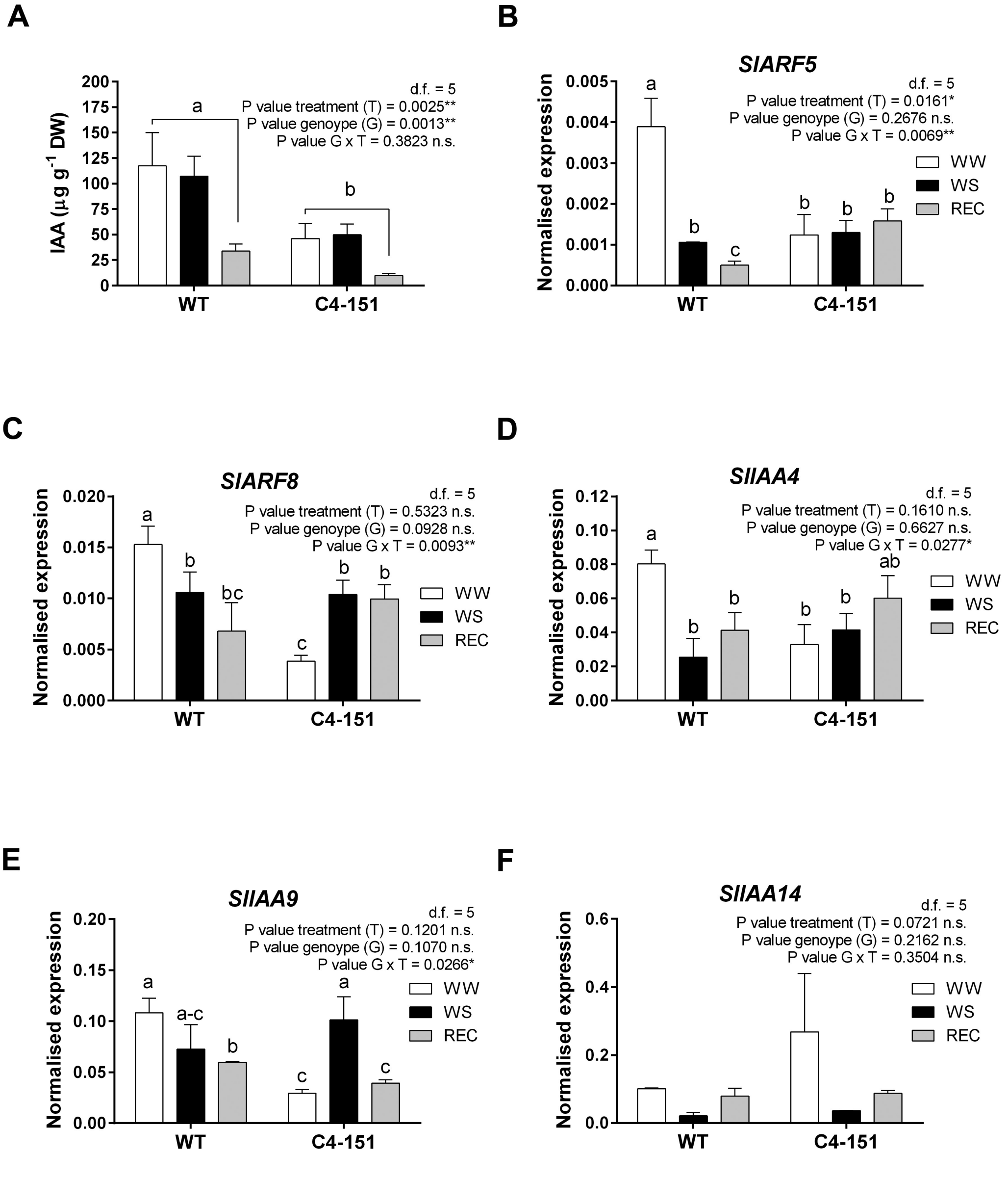

Supplement: Pagliarani_et_al_supplementary_material_R1_uhac164 [file pagliarani_et_al_supplementary_material_r1_uhac164.zip › Pagliarani_et_al_supplementary_material_R1_uhac164.pdf]
